# Safety of Daprodustat for the Treatment of Chronic Kidney Disease Anemia: Final Analysis of a Multicenter Postmarketing Surveillance Study in Japan

**DOI:** 10.1002/1744-9987.70182

**Published:** 2026-07-06

**Authors:** Tadao Akizawa, Kyomi Kanaya, Kiyomi Aizawa, Masanori Onoki, Hirofumi Ozeki, Kazuko Suzuki, Ai Hayashi, Tomohiko Asamura

**Affiliations:** ^1^ Division of Nephrology, Department of Medicine Showa Medical University Tokyo Japan; ^2^ Real‐World Data Analytics, Biostatistics, Asia Development GlaxoSmithKline K.K Tokyo Japan; ^3^ Safety Risk Evaluation, Clinical Safety & Pharmacovigilance GlaxoSmithKline K.K Tokyo Japan; ^4^ Specialty Medical Affairs, Specialty Medical Affairs and Development, Japan Medical Affairs GlaxoSmithKline K.K Tokyo Japan; ^5^ Safety Evaluation & Risk Management, Clinical Safety & Pharmacovigilance GlaxoSmithKline K.K Tokyo Japan; ^6^ Real‐World Evidence & Health Outcome Research, Japan Medical Affairs GlaxoSmithKline K.K Tokyo Japan

**Keywords:** anemia, chronic kidney disease, daprodustat, postmarketing surveillance study

## Abstract

**Introduction:**

This final analysis of a multicenter, prospective postmarketing surveillance study evaluated the safety of daprodustat in patients with chronic kidney disease anemia in routine clinical practice in Japan.

**Methods:**

Patients who initiated daprodustat between September 2020 and July 2022 were registered. The observation period was 52 weeks from treatment start or until discontinuation to assess overall safety, with an additional 52‐week follow‐up for cancer‐related events.

**Results:**

Of 1634 patients in the safety analysis set, adverse drug reactions (ADRs) occurred in 5.6% (4.8% not undergoing dialysis; 6.9% undergoing peritoneal dialysis; 6.6% undergoing hemodialysis) and serious ADRs in 2.2%. ADRs related to thromboembolism, hypertension, cardiovascular events, cancer, and retinal hemorrhage were reported in less than 1%, with no progression of autosomal dominant polycystic kidney disease. During follow‐up, no increase in cancer‐related events was observed.

**Conclusions:**

No new safety concerns were identified for daprodustat in the real‐world setting in Japan.

**Trial Registration:** GSK study: 214119

## 1 Introduction

In Japan, chronic kidney disease (CKD) affects about one in five adults, especially older adults [1]. Data from the Japanese Society for Dialysis Therapy Renal Data Registry (JRDR) show 337 414 chronic dialysis patients at the end of 2024 (2725 per million) [2]. Compared with North America and Europe, Japan relies far more on hemodialysis (over 90% of renal replacement therapy), while peritoneal dialysis (PD) (including combined cases) is only about 3% [1].

Anemia affects about 40%–50% of CKD patients and worsens with advancing stage [3, 4] It stems from reduced renal erythropoietin, impaired hematopoiesis, shorter red blood cell lifespan, disordered iron metabolism (absolute/functional deficiency), chronic bleeding (e.g., dialysis, gastrointestinal), and malnutrition or vitamin deficits [5]. Anemia markedly increases risks of cardiovascular (CV) events, mortality, hospitalization, CKD progression, and reduces quality of life and work productivity [6, 7, 8, 9].

Injectable erythropoiesis‐stimulating agent (ESA) is the standard medication for treating anemia in CKD patients, and while it is effective in avoiding blood transfusions and improving symptoms, treatment strategies aiming to achieve hemoglobin levels exceeding the guideline‐recommended range using injectable ESAs have been reported to be associated with increased risk of CV and thromboembolic events; furthermore, no improvement in overall survival or major CV events has been demonstrated; additionally, an increase in mortality, CV events, and hospitalization risk has been reported as a challenge in patients resistant to ESA [10, 11, 12, 13, 14].

Hypoxia‐inducible factor prolyl hydroxylase inhibitors (HIF‐PHIs) are oral agents that enhance erythropoiesis by activating the body's adaptive response to hypoxia, thereby helping treat anemia and maintain hemoglobin within target ranges. Five HIF‐PHIs—daprodustat, roxadustat, vadadustat, enarodustat, and molidustat—are approved in Japan, mainly for CKD‐related anemia. Considering their mechanisms, preclinical findings, and adverse events (AEs) seen in trials, the Japanese Society of Nephrology has issued guidance for their use. The guidelines highlight the need to screen and monitor for thromboembolism, hypertension, cancer, retinal hemorrhage, autosomal dominant polycystic kidney disease (ADPKD), heart failure/pulmonary hypertension, vascular calcification, hyperkalemia, liver dysfunction, and CV complications linked to abnormal glucose and lipid metabolism [15, 16, 17, 18]^.^

The safety and efficacy of daprodustat for CKD‐related anemia were confirmed in three Phase 3 studies in Japan [19, 20, 21, 22], which enrolled patients on hemodialysis (HD), PD, and not on dialysis (ND). Hemoglobin improvements with daprodustat were similar to those with injectable ESAs, and AE profiles were comparable. A pooled analysis from the Japanese Phase 3 program [23] showed comparable incidence of thromboembolic, CV, retinal, and cancer‐related AEs between daprodustat and injectable ESAs, and importantly no new safety concerns were identified.

Daprodustat received approval in Japan in June 2020 for the treatment of anemia associated with CKD. As part of the approval process, post‐marketing surveillance was mandated to evaluate its safety in real‐world clinical settings. Interim findings from this surveillance, published in 2024, revealed no new safety concerns related to daprodustat use in Japan [24]. However, these results were based on data from approximately half of the patients expected in the final analysis. The interim report also emphasized the need for further monitoring and follow‐up regarding cancer‐related events. This final analysis aims to provide comprehensive post‐marketing surveillance data on patients treated with daprodustat in Japan, covering the period up to June 2025.

## 2 Materials and Methods

### 2.1 Study Design and Patient Population

Between September 2020 and July 2022, patients in Japan who began treatment with daprodustat were enrolled in a prospective, observational, postmarketing surveillance study (GSK Study 214119). Registration was conducted via an electronic data capture (EDC) system within 14 days of initiating daprodustat therapy. The observation period lasted for 52 weeks from the start of treatment or until daprodustat was discontinued, whichever occurred first, to evaluate overall safety. Following the observation period (or after daprodustat discontinuation), patients were monitored for an additional 52 weeks to assess cancer‐related events. The study aimed to recruit 1500 patients, including at least 100 individuals undergoing PD, from approximately 200 sites. This was designed to achieve a target population of 1300 patients (as the population in the safety analysis set, accounting for potential dropouts), sufficient to identify unexpected AEs with a frequency of ≥ 0.25% at a 95% confidence level. The results presented here are based on a final analysis that includes observational data collected for up to 52 weeks and follow‐up data on cancer‐related events for an additional 52 weeks, reported as of June 2025.

The study protocol underwent review by the Pharmaceuticals and Medical Devices Agency (PMDA) and received approval from the Ethics Review Board of Kitamachi Clinic, which acted as the central ethics committee. The study was carried out in accordance with the Ministry of Health, Labour and Welfare (MHLW) Ordinance No. 171, issued on December 20, 2004, which establishes Good Postmarketing Surveillance Practice (GPSP) guidelines for conducting postmarketing surveillance and studies on pharmaceuticals. Before participation, all patients provided written informed consent, and no personally identifiable data was collected during the study.

### 2.2 Data Collection and Analyses

An EDC system was utilized for patient enrollment and data collection. The collected data included baseline patient characteristics, medical history, prior and concurrent treatment, CKD stage, dialysis status, daprodustat dosing details, and laboratory values such as hemoglobin levels. For variables permitting multiple responses (e.g., causes of anemia), each cause was coded as a binary variable (present/absent) for each patient; percentages in tables are calculated using the total number of patients as the denominator, so the sum of responses may exceed the total number of patients. AEs reported subsequent to daprodustat initiation were gathered irrespective of their suspected relationship to the drug. To summarize and report AEs and adverse drug reactions (ADRs), terminology from the Medical Dictionary for Regulatory Activities/Japanese Edition (MedDRA/J; version 27.1) was applied, categorizing them by system organ class and preferred terms. ADRs comprised events determined by the research team or investigator to have a causal relationship with daprodustat, along with those not definitively classified as unrelated to the drug. Specific ADRs of interest included thromboembolism, hypertension, CV events (excluding thromboembolism), cancer, disease progression in patients with ADPKD, and retinal hemorrhage. For these ADRs of special interest, the incidence rate was calculated per 100 patient‐years, accompanied by the exact 95% Poisson confidence interval (CI) based on the *χ* ^2^ distribution.

The safety analysis included all patients irrespective of their dialysis status during the observation period. Patient follow‐up continued until either drug discontinuation/termination or the end of the observation period (Week 52). Additionally, follow‐up for cancer‐related events extended for another 52 weeks, with a maximum duration of up to 2 years. This final analysis presents the data available as of June 2025.

The ND, PD, and HD subgroups consist of patients categorized at the study's initiation. If a patient transitioned to a different therapy (e.g., ND to PD or HD; PD to HD; or HD to ND due to renal transplantation) during the observation or follow‐up period, any ADRs reported after the transition were excluded from the original subgroup assigned at baseline. However, these ADRs were still accounted for in the overall safety analysis population.

Multivariate analysis was conducted using a logistic regression model to identify baseline risk factors for ADRs. Variables used in the analysis were determined based on the proportion of missing data and the imbalance in case numbers between subgroups. The adjusted odds ratios for AEs based on patient background factors were verified through multivariate analysis for factors meeting the criteria of “the asymptotic 95% CI does not cross 1, and the point estimate exceeds 2 or is less than 0.5.”

## 3 Results

### 3.1 Patient Disposition

The study involved 1674 registered patients, of whom 1652 either completed the 52‐week observation period or discontinued daprodustat during that period; their data was finalized for inclusion in the analysis. Eighteen patients were excluded from the safety analysis set due to specific circumstances: 11 were enrolled outside the allowable registration period, three did not attend any follow‐up visits after starting daprodustat, two received care outside the contractual timeframe, and two for whom eCRFs were inappropriately submitted by a site staff member after the principal investigator passed away. Consequently, 1634 patients were included in the safety analysis set (Figure 1a). This group comprised 1048 individuals (64.1%) in the ND group, 116 (7.1%) in the PD group and 470 (28.8%) in the HD group. An additional subset of 1406 patients completed an extended follow‐up period of up to 52 weeks, which focused on monitoring cancer‐related outcomes (Figure 1b).

**FIGURE 1:**
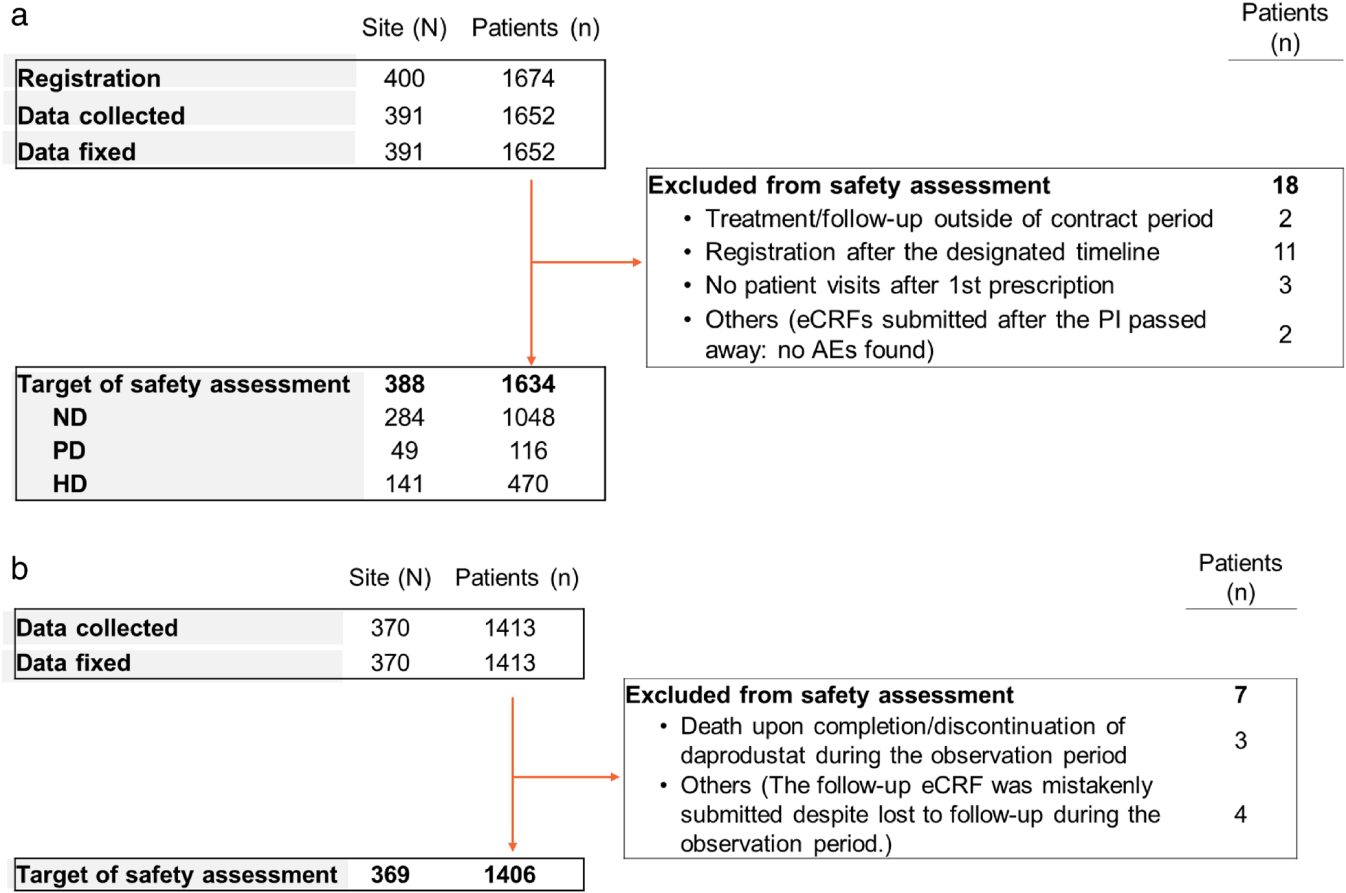
(a) Patient disposition (patients observed for up to 52‐week after daprodustat administration) and (b) patient disposition (patients followed up for up‐to‐52‐week after end of observation period). AE, adverse event; eCRF; electronic case report form; HD, hemodialysis; ND, no dialysis; PI, principal investigator; PD, peritoneal dialysis.

### 3.2 Patient Baseline Characteristics

The average age of the study population was 74.8 years (standard deviation [SD]: 12.9), with 80.9% (*n* = 1322) of patients aged 65 or older (Table 1). Men accounted for slightly more than half of the cohort (54.0%, *n* = 882). During the study, 22 patients transitioned their dialysis modality, either from PD to HD or vice versa. The main comorbidities were hypertension (82.3%), CV events (excluding thromboembolism) (23.4%), thromboembolism (16.6%), and heart failure (15.2%). Before starting daprodustat, the majority of patients (89.4%) had been treated for CKD‐related anemia, including 729 patients (44.6%, *n* = 729/1634) who received injectable ESAs, 33.8% (*n* = 354/1048) in the ND group, 72.4% (*n* = 84/116) in the PD group, and 61.9% (*n* = 291/470) in the HD group. Among those previously treated with injectable ESAs, darbepoetin alfa was the most commonly used agent (*n* = 364), followed by epoetin beta pegol (*n* = 296) and epoetin (*n* = 69) (Table 2). Additionally, 221 patients (13.5%) received iron therapy, including iron supplements or iron‐containing phosphate binders, before the study began, with corresponding proportions by dialysis group being 11.9% (*n* = 125/1048) in the ND group, 16.4% (*n* = 19/116) in the PD group, and 16.4% (*n* = 77/470) in the HD group. Regarding iron therapy, 431 patients (26.4%) received concomitant iron therapy, distributed as follows: 21.7% (*n* = 227/1048) in the ND group, 38.8% (*n* = 45/116) in the PD group, and 33.8% (159/470) in the HD group.

**TABLE 1:** Baseline patient characteristics.

Characteristic, *n* (%)	Safety population (*N* = 1634)	Dialysis status at baseline		
		ND (*N* = 1048)	PD (*N* = 116)	HD (*N* = 470)
Gender				
Male	882 (54.0)	551 (48.8)	72 (62.1)	299 (63.6)
Female	752 (46.0)	537 (51.2)	44 (37.9)	171 (36.4)
Age				
< 15 years	0 (0.0)	0 (0.0)	0 (0.0)	0 (0.0)
≥ 15–< 65 years	312 (19.1)	139 (13.3)	49 (42.2)	124 (26.4)
≥ 65 years	1322 (80.9)	909 (86.7)	67 (57.8)	346 (73.6)
Cause of anemia^a^				
DKD	568 (34.8)	342 (32.6)	44 (37.9)	182 (38.7)
Chronic glomerular				
Nephritis (CGN)	267 (16.3)	123 (11.7)	29 (25.0)	115 (24.5)
Nephrosclerosis	565 (34.6)	426 (40.6)	25 (21.6)	114 (24.3)
ADPKD	58 (3.5)	31 (3.0)	6 (5.2)	21 (4.5)
Other	273 (16.7)	199 (19.0)	18 (15.5)	56 (11.9)
CKD anemia duration				
≤ 2 years	293 (17.9)	213 (20.3)	22 (19.0)	58 (12.3)
> 2–≤ 5 years	332 (20.3)	201 (19.2)	22 (19.0)	109 (23.2)
> 5–≤ 10 years	252 (15.4)	134 (12.8)	19 (16.4)	99 (21.1)
> 10 years	388 (23.7)	201 (19.2)	34 (29.3)	153 (32.6)
Unknown	369 (22.6)	299 (28.5)	19 (16.4)	51 (10.9)
Body weight				
< 55 kg	720 (44.1)	458 (43.7)	37 (31.9)	225 (47.9)
≥ 55 kg	723 (44.2)	413 (39.4)	77 (66.4)	233 (49.6)
Unknown	191 (11.7)	177 (16.9)	2 (1.7)	12 (2.6)
BMI				
< 18.5 kg/m^2^	202 (12.4)	113 (10.8)	7 (6.0)	82 (17.4)
≥ 18.5–< 25 kg/m^2^	864 (52.9)	532 (50.8)	67 (57.8)	265 (56.4)
≥ 25 kg/m^2^	306 (18.7)	183 (17.5)	39 (33.6)	84 (17.9)
Unknown	262 (16.0)	220 (21.0)	3 (2.6)	39 (8.3)
Medical history^a^ ^,^ ^b^				
Thromboembolism	90 (5.5)	36 (3.4)	11 (9.5)	43 (9.1)
Hypertension	11 (0.7)	7 (0.7)	2 (1.7)	2 (0.4)
Cardiovascular events	53 (3.2)	28 (2.7)	6 (5.2)	19 (4.0)
Cancer	87 (5.3)	54 (5.2)	3 (2.6)	30 (6.4)
Retinal hemorrhage	11 (0.7)	1 (0.1)	0 (0.0)	10 (2.1)
Hepatic dysfunction	17 (1.0)	12 (1.1)	2 (1.7)	3 (0.6)
Heart failure	10 (0.6)	6 (0.6)	1 (0.9)	3 (0.6)
Other	257 (15.7)	123 (11.7)	20 (17.2)	114 (24.3)
Comorbidities^a^ ^,^ ^c^				
Thromboembolism	271 (16.6)	156 (14.9)	19 (16.4)	96 (20.4)
Hypertension	1345 (82.3)	868 (82.8)	100 (86.2)	377 (80.2)
Cardiovascular events	382 (23.4)	256 (24.4)	29 (25.0)	97 (20.6)
Cancer	61 (3.7)	38 (3.6)	4 (3.4)	19 (4.0)
Retinal hemorrhage	31 (1.9)	20 (1.9)	3 (2.6)	8 (1.7)
Hepatic dysfunction	83 (5.1)	56 (5.3)	6 (5.2)	21 (4.5)
Heart failure	249 (15.2)	178 (17.0)	24 (20.7)	47 (10.0)
Other	1628 (99.6)	1046 (99.8)	115 (99.1)	467 (99.4)
History of renal transplant	55 (3.4)	33 (3.1)	7 (6.0)	15 (3.2)
History of nephrectomy	38 (2.3)	16 (1.5)	2 (1.7)	20 (4.3)
Number of kidneys				
0	6 (0.4)	0 (0.0)	0 (0.0)	6 (1.3)
1	22 (1.3)	9 (0.9)	1 (0.9)	12 (2.6)
2	1606 (98.3)	1039 (99.1)	115 (99.1)	452 (96.2)
Any prior treatment				
Yes	1460 (89.4)	914 (87.2)	108 (93.1)	438 (93.2)
No	174 (10.6)	134 (12.8)	8 (6.9)	32 (6.8)
Prior treatments^a^				
Iron supplements	170 (10.4)	113 (10.8)	9 (7.8)	48 (10.2)
Phosphate binders containing iron	57 (3.5)	13 (1.2)	10 (8.6)	34 (7.2)
Any iron^d^	221 (13.5)	125 (11.9)	19 (16.4)	77 (16.4)
HIF‐PHI	78 (4.8)	26 (2.5)	7 (6.0)	45 (9.6)
Injectable ESA	729 (44.6)	354 (33.8)	84 (72.4)	291 (61.9)
ESA and/or any iron^d^, ^e^	840 (51.4)	429 (40.9)	89 (76.7)	322 (68.5)
HIF‐PHI and/or any iron^d^, ^f^	286 (17.5)	149 (14.2)	22 (19.0)	115 (24.5)
Concomitant iron^d^	431 (26.4)	227 (21.7)	45 (38.8)	159 (33.8)
Baseline hemoglobin				
< 9 g/dL	281 (17.2)	175 (16.7)	21 (18.5)	85 (18.1)
≥ 9–< 10 g/dL	462 (28.3)	313 (29.9)	19 (16.4)	130 (27.7)
≥ 10–< 11 g/dL	492 (30.1)	337 (32.2)	29 (25.0)	126 (26.8)
≥ 11–< 12 g/dL	248 (15.2)	134 (12.8)	34 (29.3)	80 (17.0)
≥ 12–< 13 g/dL	112 (6.9)	63 (6.0)	9 (7.8)	40 (8.5)
≥ 13 g/dL	27 (1.7)	14 (1.3)	4 (3.4)	9 (1.9)
Unknown	12 (0.7)	12 (1.1)	0 (0.0)	0 (0.0)
Baseline SBP				
< 130 mmHg	571 (34.9)	441 (42.1)	35 (30.2)	95 (20.2)
≥ 130 mmHg	997 (61.0)	543 (51.8)	81 (69.8)	373 (79.4)
Unknown	66 (4.0)	64 (6.1)	0 (0.0)	2 (0.4)
Baseline DBP				
< 80 mmHg	1078 (66.0)	740 (70.6)	63 (54.3)	275 (58.5)
≥ 80 mmHg	490 (30.0)	244 (23.3)	53 (45.7)	193 (41.1)
Unknown	66 (4.0)	64 (6.1)	0	2 (0.4)
Baseline eGFR, mL/min/1.73 m^2^				
< 15	583 (35.7)	263 (25.1)	N/A	N/A
≥ 15–< 30	407 (24.9)	404 (38.5)	N/A	N/A
≥ 30–< 60	294 (18.0)	294 (28.1)	N/A	N/A
≥ 60	15 (0.9)	15 (1.4)	N/A	N/A
Unknown	335 (20.5)	72 (6.9)	N/A	N/A

*Note:* The sum of *n* across items may exceed *N* because multiple responses were allowed.

Abbreviations: ADPKD, autosomal dominant polycystic kidney disease; BMI, body mass index; CGN, chronic glomerulonephritis; CKD, chronic kidney disease; DBP, diastolic blood pressure; DKD, diabetic kidney disease; eGRF, estimated glomerular filtration rate; ESA, erythropoiesis‐stimulating agent; HD, hemodialysis; HIF‐PHI, hypoxia‐inducible factor prolyl hydroxylase inhibitor; N/A, not applicable; ND, no dialysis; PD, peritoneal dialysis; SBP, systolic blood pressure.

^a^ Patients could report multiple items. Values are *n* (number of patients reporting the item); percentages are % of all patients (*N* = 1634 (overall), 1048 (ND), 116 (PD), 470 (HD)).

^b^ Resolved prior to initiation of daprodustat.

^c^ Ongoing when initiating daprodustat.

^d^ Iron supplements or phosphate binders containing iron.

^e^ “ESA and/or any iron” includes patients who received ESA, iron, or both prior to daprodustat initiation.

^f^ “HIF‐PHI and/or any iron” includes patients who received HIF‐PHI, iron, or both prior to daprodustat initiation.

**TABLE 2:** Prior treatment with and without injectable ESAs.

Characteristic, *n* (%)	Safety population (*N* = 1634)	Dialysis status at baseline		
		ND (*n* = 1048)	PD (*n* = 116)	HD (*n* = 470)
Patients with prior ESA	729	354	84	291
Prior ESA				
Epoetin	69 (9.5)	1 (0.3)	0 (0.0)	68 (23.4)
Epoetin beta pegol	296 (40.6)	204 (57.6)	61 (72.6)	31 (10.7)
Darbepoetin alfa	364 (49.9)	149 (42.1)	23 (27.4)	192 (66.0)
ESA dose (IU/week)				
< 4500	294 (40.3)	213 (60.1)	29 (34.5)	52 (17.0)
≥ 4500–< 6000	81 (11.1)	31 (8.8)	11 (13.1)	39 (13.4)
≥ 6000	354 (48.6)	110 (31.1)	44 (52.4)	200 (68.7)
ERI (IU/week/kg/g/dL)				
< 5.7	185 (25.4)	118 (33.3)	24 (28.6)	43 (14.8)
≥ 5.7–< 9.7	140 (19.2)	77 (21.8)	16 (19.0)	47 (16.2)
≥ 9.7–< 15.5	120 (16.5)	45 (12.7)	19 (22.6)	56 (19.2)
≥ 15.5	235 (32.2)	70 (19.8)	23 (27.4)	142 (48.8)
Unknown	49 (6.7)	44 (12.4)	2 (2.4)	3 (1.0)
Insufficient effectiveness of prior ESA treatment^a^	330 (45.3)	163 (46.0)	23 (27.3)	144 (49.5)
Baseline Hgb in patients with prior ESA treatment (g/dL)				
< 9 g/dL	118 (16.2)	49 (13.8)	15 (17.9)	54 (18.6)
≥ 9–< 10 g/dL	174 (23.9)	79 (22.3)	16 (19.0)	79 (27.1)
≥ 10–< 11 g/dL	214 (29.4)	113 (31.9)	19 (22.6)	82 (28.2)
≥ 11–< 12 g/dL	141 (19.3)	72 (20.3)	24 (28.6)	45 (15.5)
≥ 12–< 13 g/dL	64 (8.8)	32 (9.0)	7 (8.3)	25 (8.6)
≥ 13 g/dL	17 (2.3)	8 (2.3)	3 (3.6)	6 (2.1)
Unknown	1 (0.1)	1 (0.3)	0 (0.0)	0 (0.0)
Patients without prior ESA	905	694	32	179
Baseline Hgb in patients without prior ESA treatment (g/dL)				
< 9 g/dL	163 (18.0)	126 (18.2)	6 (18.8)	31 (17.3)
≥ 9–< 10 g/dL	288 (31.8)	234 (33.7)	3 (9.4)	51 (28.5)
≥ 10–< 11 g/dL	278 (30.7)	224 (32.3)	10 (31.3)	44 (24.6)
≥ 11–< 12 g/dL	107 (11.8)	62 (8.9)	10 (31.3)	35 (19.6)
≥ 12–< 13 g/dL	48 (5.3)	31 (4.5)	2 (6.3)	15 (8.4)
≥ 13 g/dL	10 (1.1)	6 (0.9)	1 (3.1)	3 (1.7)
Unknown	11 (1.2)	11 (1.6)	0 (0.0)	0 (0.0)

Abbreviations: ERI, ESA resistance index; ESA, erythropoietin‐stimulating agent; HD, hemodialysis; Hgb, hemoglobin; ND, no dialysis; PD, peritoneal dialysis.

^a^ “Insufficient effectiveness of prior ESA treatment” was a category recorded in the CRF and based on the investigator's clinical judgment.

### 3.3 Daprodustat Exposure

The initial daily dose of daprodustat (mean [SD]/median) was 3.3 [1.2]/4.0 mg overall, 3.0 [1.3]/2.0 mg in the ND group, 3.6 [0.9]/4.0 mg in the PD group, and 3.9 [0.8]/4.0 mg in the HD group. During the observation period, the daily dose (mean [SD]/median) was 4.0 [2.5]/4.0 mg overall, 3.3 [1.8]/2.9 mg in the ND group, 4.9 [2.4]/4.0 mg in the PD group, and 5.4 [3.0]/4.3 mg in the HD group. Twenty‐four patients received an initial dose of daprodustat exceeding the recommended maximum starting dose of 4 mg, including 15 from the ND group, one from the PD group, and eight from the HD group; An ADR (breast cancer, serious, onset 646 days after daprodustat initiation, outcome unknown) was reported in one patient from the ND group. On the other hand, no patients were administered daprodustat exceeding the maximum recommended daily dose of 24 mg. Trends in daprodustat dosing over time, categorized by dialysis status and prior treatment with or without injectable ESAs, are presented in Figure S1.

### 3.4 ADRs

ADRs were observed in 5.6% of the overall study population (*n* = 91/1634). When analyzed by dialysis subgroup, the incidence of ADRs was 4.8% (*n* = 50/1048) in the ND group, 6.9% (*n* = 8/116) in the PD group, and 6.6% (*n* = 31/470) in the HD group (Table 3). The most frequently reported ADRs included hypertension (0.5%, *n* = 8) and nausea (0.4%, *n* = 7). Serious ADRs were identified in 2.2% of the population (*n* = 36/1634), with the main ones being nephrogenic anemia (0.3%, *n* = 5) and colon cancer (0.2%, *n* = 3). Among the reported deaths (*n* = 12), 10 patients (“death” [*n* = 2], “metastases to liver,” “kidney cancer,” and “metastases to lymph node,” “lymphoma,” the following events in a single patient (“colon cancer,” “metastases to liver,” “metastases to peritoneal,” “metastases to lung,” “cerebral infarction” and “abdominal pain”), “lung adenocarcinoma” and “metastases to bone,” “aortic dissection,” “acute myocardial infarction,” “pulmonary tuberculosis” [each *n* = 1]) belonged to the ND group, and two patients (“metastases to bone,” “heart failure” [each *n* = 1]) belonged to the HD group.

**TABLE 3:** ADRs after initiation of daprodustat treatment.^a^

ADR, *n* (%)	Safety population (*N* = 1634)		ND (*n* = 1048)		PD (*n* = 116)		HD (*n* = 470)	
	Any	Serious	Any	Serious	Any	Serious	Any	Serious
Any ADR	91 (5.6)	36 (2.2)	50 (4.8)	22 (2.1)	8 (6.9)	1 (0.9)	31 (6.6)	12 (2.6)
Investigations	14 (0.9)	0 (0.0)	6 (0.6)	0 (0.0)	3 (2.6)	0 (0.0)	5 (1.1)	0 (0.0)
Hgb decreased	5 (0.3)	0 (0.0)	1 (0.1)	0 (0.0)	1 (0.9)	0 (0.0)	3 (0.6)	0 (0.0)
Hgb increased	5 (0.3)	0 (0.0)	1 (0.1)	0 (0.0)	2 (1.7)	0 (0.0)	2 (0.4)	0 (0.0)
Blood iron decreased	1 (0.1)	0 (0.0)	1 (0.1)	0 (0.0)	0 (0.0)	0 (0.0)	0 (0.0)	0 (0.0)
BP increased	1 (0.1)	0 (0.0)	1 (0.1)	0 (0.0)	0 (0.0)	0 (0.0)	0 (0.0)	0 (0.0)
Fibrin and fibrinogen degradation product increased	1 (0.1)	0 (0.0)	1 (0.1)	0 (0.0)	0 (0.0)	0 (0.0)	0 (0.0)	0 (0.0)
Mean corpuscular volume decreased	1 (0.1)	0 (0.0)	1 (0.1)	0 (0.0)	0 (0.0)	0 (0.0)	0 (0.0)	0 (0.0)
Platelets decreased	1 (0.1)	0 (0.0)	0 (0.0)	0 (0.0)	0 (0.0)	0 (0.0)	1 (0.2)	0 (0.0)
Transferrin saturation decreased	1 (0.1)	0 (0.0)	1 (0.1)	0 (0.0)	0 (0.0)	0 (0.0)	0 (0.0)	0 (0.0)
GI disorders	13 (0.8)	2 (0.1)	5 (0.5)	1 (0.1)	1 (0.9)	0 (0.0)	7 (1.5)	2 (0.4)
Nausea	7 (0.4)	0 (0.0)	2 (0.2)	0 (0.0)	1 (0.9)	0 (0.0)	4 (0.9)	0 (0.0)
Abdominal pain	2 (0.1)	1 (0.1)	1 (0.1)	1 (0.1)	0 (0.0)	0 (0.0)	1 (0.2)	0 (0.0)
Diarrhea	2 (0.1)	0 (0.0)	0 (0.0)	0 (0.0)	0 (0.0)	0 (0.0)	2 (0.4)	0 (0.0)
Abdominal discomfort	1 (0.1)	0 (0.0)	0 (0.0)	0 (0.0)	0 (0.0)	0 (0.0)	1 (0.2)	0 (0.0)
Acute abdomen	1 (0.1)	1 (0.1)	0 (0.0)	0 (0.0)	0 (0.0)	0 (0.0)	1 (0.2)	1 (0.2)
Gastritis	1 (0.1)	0 (0.0)	1 (0.1)	0 (0.0)	0 (0.0)	0 (0.0)	0 (0.0)	0 (0.0)
Diverticular perforation	1 (0.1)	1 (0.1)	0 (0.0)	0 (0.0)	0 (0.0)	0 (0.0)	1 (0.2)	1 (0.2)
General disorders and administration site conditions	13 (0.8)	3 (0.2)	6 (0.4)	2 (0.2)	2 (1.7)	0 (0.0)	5 (1.1)	1 (0.2)
Condition aggravated	5 (0.3)	1 (0.1)	2 (0.2)	0 (0.0)	0 (0.0)	0 (0.0)	3 (0.6)	1 (0.2)
Death	2 (0.1)	2 (0.1)	2 (0.2)	2 (0.2)	0 (0.0)	0 (0.0)	0 (0.0)	0 (0.0)
Malaise	2 (0.1)	0 (0.0)	0 (0.0)	0 (0.0)	1 (0.9)	0 (0.0)	1 (0.2)	0 (0.0)
Feeling abnormal	1 (0.1)	0 (0.0)	0 (0.0)	0 (0.0)	1 (0.9)	0 (0.0)	0 (0.0)	0 (0.0)
Pain	1 (0.1)	0 (0.0)	0 (0.0)	0 (0.0)	0 (0.0)	0 (0.0)	1 (0.3)	0 (0.0)
Pyrexia	1 (0.1)	0 (0.0)	1 (0.1)	0 (0.0)	0 (0.0)	0 (0.0)	0 (0.0)	0 (0.0)
Thirst	1 (0.1)	0 (0.0)	1 (0.1)	0 (0.0)	0 (0.0)	0 (0.0)	0 (0.0)	0 (0.0)
Vascular disorders	12 (0.7)	3 (0.2)	9 (0.9)	3 (0.3)	0 (0.0)	0 (0.0)	2 (0.4)	0 (0.0)
Hypertension	8 (0.5)	0 (0.0)	5 (0.5)	0 (0.0)	0 (0.0)	0 (0.0)	2 (0.4)	0 (0.0)
DVT	2 (0.1)	2 (0.1)	2 (0.2)	2 (0.2)	0 (0.0)	0 (0.0)	0 (0.0)	0 (0.0)
Aortic dissection	1 (0.1)	1 (0.1)	1 (0.1)	1 (0.1)	0 (0.0)	0 (0.0)	0 (0.0)	0 (0.0)
Hot flashes	1 (0.1)	0 (0.0)	1 (0.1)	0 (0.0)	0 (0.0)	0 (0.0)	0 (0.0)	0 (0.0)
Blood and lymphatic system disorders	11 (0.7)	6 (0.4)	4 (0.4)	2 (0.2)	0 (0.0)	0 (0.0)	7 (1.5)	4 (0.9)
Nephrogenic anemia	5 (0.3)	5 (0.3)	2 (0.2)	2 (0.2)	0 (0.0)	0 (0.0)	3 (0.6)	3 (0.6)
Anemia	3 (0.2)	0 (0.0)	0 (0.0)	0 (0.0)	0 (0.0)	0 (0.0)	3 (0.6)	0 (0.0)
Iron deficiency nemia	3 (0.2)	0 (0.0)	2 (0.2)	0 (0.0)	0 (0.0)	0 (0.0)	1 (0.2)	0 (0.0)
Pancytopenia	1 (0.1)	1 (0.1)	0 (0.0)	0 (0.0)	0 (0.0)	0 (0.0)	1 (0.2)	1 (0.2)
Neoplasms^b^	11 (0.7)	11 (0.7)	9 (0.9)	9 (0.9)	0 (0.0)	0 (0.0)	2 (0.4)	1 (0.2)
Colon cancer	3 (0.2)	3 (0.2)	2 (0.2)	2 (0.2)	0 (0.0)	0 (0.0)	1 (0.2)	1 (0.2)
Lung adenocarcinoma	2 (0.1)	2 (0.1)	2 (0.2)	2 (0.2)	0 (0.0)	0 (0.0)	0 (0.0)	0 (0.0)
Metastases to bone	2 (0.1)	2 (0.1)	1 (0.1)	1 (0.1)	0 (0.0)	0 (0.0)	1 (0.2)	1 (0.3)
Metastases to liver	2 (0.1)	2 (0.1)	2 (0.2)	2 (0.2)	0 (0.0)	0 (0.0)	0 (0.0)	0 (0.0)
Breast cancer	1 (0.1)	1 (0.1)	1 (0.1)	1 (0.1)	0 (0.0)	0 (0.0)	0 (0.0)	0 (0.0)
Gastric cancer	1 (0.1)	1 (0.1)	1 (0.1)	1 (0.1)	0 (0.0)	0 (0.0)	0 (0.0)	0 (0.0)
Lymphoma	1 (0.1)	1 (0.1)	1 (0.1)	1 (0.1)	0 (0.0)	0 (0.0)	0 (0.0)	0 (0.0)
Marrow hyperplasia	1 (0.1)	0 (0.0)	0 (0.0)	0 (0.0)	0 (0.0)	0 (0.0)	1 (0.2)	0 (0.0)
Metastases to lung	1 (0.1)	1 (0.1)	1 (0.1)	1 (0.1)	0 (0.0)	0 (0.0)	0 (0.0)	0 (0.0)
Metastases to lymph node	1 (0.1)	1 (0.1)	1 (0.1)	1 (0.1)	0 (0.0)	0 (0.0)	0 (0.0)	0 (0.0)
Pancreatic cancer	1 (0.1)	1 (0.1)	1 (0.1)	1 (0.1)	0 (0.0)	0 (0.0)	0 (0.0)	0 (0.0)
Kidney cancer	1 (0.1)	1 (0.1)	1 (0.1)	1 (0.1)	0 (0.0)	0 (0.0)	0 (0.0)	0 (0.0)
Transitional cell carcinoma	1 (0.1)	1 (0.1)	1 (0.1)	1 (0.1)	0 (0.0)	0 (0.0)	0 (0.0)	0 (0.0)
Metastases to peritoneal	1 (0.1)	1 (0.1)	1 (0.1)	1 (0.1)	0 (0.0)	0 (0.0)	0 (0.0)	0 (0.0)
Skin and subcutaneous tissue disorders	8 (0.5)	0 (0.0)	7 (0.7)	0 (0.0)	1 (0.9)	0 (0.0)	0 (0.0)	0 (0.0)
Drug eruption	3 (0.2)	0 (0.0)	2 (0.2)	0 (0.0)	1 (0.9)	0 (0.0)	0 (0.0)	0 (0.0)
Pruritus	2 (0.1)	0 (0.0)	2 (0.2)	0 (0.0)	0 (0.0)	0 (0.0)	0 (0.0)	0 (0.0)
Rash	1 (0.1)	0 (0.0)	1 (0.2)	0 (0.0)	0 (0.0)	0 (0.0)	0 (0.0)	0 (0.0)
Eruption	1 (0.1)	0 (0.0)	1 (0.2)	0 (0.0)	0 (0.0)	0 (0.0)	0 (0.0)	0 (0.0)
Urticaria	1 (0.1)	0 (0.0)	1 (0.2)	0 (0.0)	0 (0.0)	0 (0.0)	0 (0.0)	0 (0.0)
Cardiac disorders	5 (0.3)	3 (0.2)	3 (0.3)	2 (0.2)	0 (0.0)	0 (0.0)	2 (0.4)	1 (0.2)
Palpitations	2 (0.1)	0 (0.0)	1 (0.1)	0 (0.0)	0 (0.0)	0 (0.0)	1 (0.2)	0 (0.0)
Acute myocardial infarction	1 (0.1)	1 (0.1)	1 (0.1)	1 (0.1)	0 (0.0)	0 (0.0)	0 (0.0)	0 (0.0)
Cardiac failure	1 (0.1)	1 (0.1)	0 (0.0)	0 (0.0)	0 (0.0)	0 (0.0)	1 (0.2)	1 (0.2)
Cardiac failure chronic	1 (0.1)	1 (0.1)	1 (0.1)	1 (0.1)	0 (0.0)	0 (0.0)	0 (0.0)	0 (0.0)
Nervous system disorders	5 (0.3)	2 (0.1)	2 (0.2)	1 (0.1)	1 (0.9)	0 (0.0)	2 (0.4)	1 (0.2)
Cerebral infarction	2 (0.1)	2 (0.1)	1 (0.1)	1 (0.1)	0 (0.0)	0 (0.0)	1 (0.2)	1 (0.2)
Dizziness	2 (0.1)	0 (0.0)	1 (0.1)	0 (0.0)	1 (0.9)	0 (0.0)	0 (0.0)	0 (0.0)
Balance disorder	1 (0.1)	0 (0.0)	0 (0.0)	0 (0.0)	0 (0.0)	0 (0.0)	1 (0.2)	0 (0.0)
Injury, poisoning, and procedural complications	3 (0.2)	3 (0.2)	1 (0.1)	1 (0.1)	0 (0.0)	0 (0.0)	2 (0.4)	2 (0.4)
Shunt occlusion	2 (0.1)	2 (0.1)	0 (0.0)	0 (0.0)	0 (0.0)	0 (0.0)	2 (0.4)	2 (0.4)
Heat exhaustion	1 (0.1)	1 (0.1)	1 (0.1)	1 (0.1)	0 (0.0)	0 (0.0)	0 (0.0)	0 (0.0)
Eye disorders	2 (0.1)	2 (0.1)	0 (0.0)	0 (0.0)	1 (0.9)	1 (0.9)	1 (0.2)	1 (0.2)
Retinal hemorrhage	2 (0.1)	2 (0.1)	0 (0.0)	0 (0.0)	1 (0.9)	1 (0.9)	1 (0.2)	1 (0.2)
Metabolism and nutritional disorders	2 (0.1)	1 (0.1)	1 (0.1)	1 (0.1)	0 (0.0)	0 (0.0)	1 (0.2)	0 (0.0)
Dehydration	1 (0.1)	1 (0.1)	1 (0.1)	1 (0.1)	0 (0.0)	0 (0.0)	0 (0.0)	0 (0.0)
Fluid retention	1 (0.1)	0 (0.0)	0 (0.0)	0 (0.0)	0 (0.0)	0 (0.0)	1 (0.2)	0 (0.0)
Respiratory, thoracic and mediastinal disorders	2 (0.2)	1 (0.1)	0 (0.0)	0 (0.0)	0 (0.0)	0 (0.0)	1 (0.2)	0 (0.0)
Dyspnea exertional	1 (0.1)	0 (0.0)	0 (0.0)	0 (0.0)	0 (0.0)	0 (0.0)	1 (0.2)	0 (0.0)
Interstitial lung disease	1 (0.1)	1 (0.1)	0 (0.0)	0 (0.0)	0 (0.0)	0 (0.0)	0 (0.0)	0 (0.0)
Wheezing	1 (0.1)	0 (0.0)	0 (0.0)	0 (0.0)	0 (0.0)	0 (0.0)	1 (0.2)	0 (0.0)
Ear and labyrinth disorders	1 (0.1)	1 (0.1)	1 (0.1)	1 (0.1)	0 (0.0)	0 (0.0)	0 (0.0)	0 (0.0)
Deafness neurosensory	1 (0.1)	1 (0.1)	1 (0.1)	1 (0.1)	0 (0.0)	0 (0.0)	0 (0.0)	0 (0.0)
Tinnitus	1 (0.1)	0 (0.0)	1 (0.1)	0 (0.0)	0 (0.0)	0 (0.0)	0 (0.0)	0 (0.0)
Endocrine disorders	1 (0.1)	1 (0.1)	1 (0.1)	1 (0.1)	0 (0.0)	0 (0.0)	0 (0.0)	0 (0.0)
Hypothyroidism	1 (0.1)	1 (0.1)	1 (0.1)	1 (0.1)	0 (0.0)	0 (0.0)	0 (0.0)	0 (0.0)
Hepatobiliary disorders	1 (0.1)	0 (0.0)	1 (0.1)	0 (0.0)	0 (0.0)	0 (0.0)	0 (0.0)	0 (0.0)
Hepatic function abnormal	1 (0.1)	0 (0.0)	1 (0.1)	0 (0.0)	0 (0.0)	0 (0.0)	0 (0.0)	0 (0.0)
Infections and infestations	1 (0.1)	1 (0.1)	1 (0.1)	1 (0.1)	0 (0.0)	0 (0.0)	0 (0.0)	0 (0.0)
Pulmonary tuberculosis	1 (0.1)	1 (0.1)	1 (0.1)	1 (0.1)	0 (0.0)	0 (0.0)	0 (0.0)	0 (0.0)
Musculoskeletal and connective tissue disorders	1 (0.1)	0 (0.0)	0 (0.0)	0 (0.0)	0 (0.0)	0 (0.0)	1 (0.2)	0 (0.0)
Pain in extremity	1 (0.1)	0 (0.0)	0 (0.0)	0 (0.0)	0 (0.0)	0 (0.0)	1 (0.2)	0 (0.0)

*Note:* ADRs were coded using MedDRA/J; version 27.1.

Abbreviations: ADR, adverse drug reaction; AVA, arteriovenous access; BP, blood pressure; DVT, deep vein thrombosis; GI, gastrointestinal; HD, hemodialysis; Hgb, hemoglobin; ND, no dialysis; PD, peritoneal dialysis.

^a^ The safety analysis population includes ADRs reported for all patients included in the study until the end of the observation period (or follow‐up period for cancer‐related events). The ND, PD, and ND groups included ADRs reported for patients within each subgroup until they switched therapy or until the end of the observation period (or follow‐up period for cancer‐related events) if they did not switch therapy.

^b^ Benign, malignant, and unspecified (including cysts and polyps).

### 3.5 ADRs of Special Interest

ADRs related to thromboembolism were reported in 0.4% (*n* = 7/1634), including four patients (deep vein thrombosis [*n* = 2], stroke [*n* = 1], and acute myocardial infarction [*n* = 1]) in the ND group, three patients (occlusion of HD arteriovenous access (AVA) [*n* = 2] and stroke [*n* = 1]) in the HD group (Table 4). The outcomes of each event included five events of recovery or improvement and two events of death. Among the two deaths, one event of cerebral infarction (ND group) involved carotid artery occlusion as a complication. The other event of acute myocardial infarction (ND group) had a medical history of pulmonary malignancy and complications including alcoholic cirrhosis, emphysema, chronic glomerulonephritis, and benign prostatic hyperplasia. The ADR incidence rate per 100 patient‐years was 0.5/100 patient‐years.

**TABLE 4:** ADRs of special interest.

ADR, *n* (%)	Safety population (*N* = 1634)			ND (*n* = 1048)		PD (*n* = 116)		HD (*n* = 470)	
	Time to onset, *n*, median (range), days^a^	Any	Serious	Any	Serious	Any	Serious	Any	Serious
Thromboembolism	*n* = 7; 175 (7–283)	7 (0.4)	7 (0.4)	4 (0.4)	4 (0.4)	0 (0.0)	0 (0.0)	3 (0.6)	3 (0.6)
Hypertension	*n* = 9; 50 (8–207)	9 (0.6)	0 (0.0)	6 (0.6)	0 (0.0)	0 (0.0)	0 (0.0)	2 (0.4)	0 (0.0)
CV events^b^	*n* = 2; 29 (21–37)	2 (0.1)	2 (0.1)	1 (0.1)	1 (0.1)	0 (0.0)	0 (0.0)	1 (0.2)	1 (0.2)
Cancer	*n* = 11; 261 (18–646)	11 (0.7)	11 (0.7)	9 (0.9)	9 (0.9)	0 (0.0)	0 (0.0)	2 (0.4)	2 (0.4)
Progression in patients with ADPKD	—	0 (0.0)	0 (0.0)	0 (0.0)	0 (0.0)	0 (0.0)	0 (0.0)	0 (0.0)	0 (0.0)
Retinal hemorrhage	*n* = 2; 183 (105–261)	2 (0.1)	2 (0.1)	0 (0.0)	0 (0.0)	1 (0.9)	1 (0.9)	1 (0.2)	1 (0.2)
GI erosion	*n* = 1; 62 (62–62)	1 (0.1)	1 (0.1)	0 (0.0)	0 (0.0)	0 (0.0)	0 (0.0)	1 (0.2)	1 (0.2)

*Note: n* indicates the number of patients with evaluable time‐to‐onset data.

Abbreviations: ADPKD, autosomal dominant polycystic kidney disease; ADR, adverse drug reaction; CV, cardiovascular; GI, gastrointestinal; HD, hemodialysis; ND, no dialysis; PD, peritoneal dialysis.

^a^ Time to onset was calculated from daprodustat initiation to the first occurrence of each ADR of special interest and is presented as median (range) in the safety population.

^b^ Except thromboembolism.

ADRs related to hypertension were reported in 0.6% (*n* = 9/1634), including eight with hypertension and one with increased blood pressure. Of these, six patients occurred in the ND group and two patients in the HD group. None of the hypertension‐related ADRs were classified as serious, and all patients either improved or fully recovered, except for one patient with an “unknown” outcome. All 9 patients had hypertension at baseline. The ADR incidence rate per 100 patient‐years was 0.7/100 patient‐years.

ADRs related to CV events (excluding thromboembolism) were reported in 0.1% (*n* = 2/1634), including one patient of new‐onset heart failure and one patient of exacerbation of chronic heart failure, both of which were serious. The patient of heart failure (HD group) resulted in death, and this patient had no prior history of heart failure, but the dose of the previous treatment drug ESA was “6000 IU/week or more.” The recovered chronic heart failure patient (ND group) had a history of chronic heart failure at baseline, and the dose of the previous treatment drug ESA was “6000 IU/week or more” (Table S1). The ADR incidence rate per 100 patient‐years was 0.2/100 patient‐years.

ADRs associated with cancer were reported in 0.7% (*n* = 11/1634), with all patients classified as serious. The breakdown is as follows: “colon cancer” − 3 patents (0.2%), “lung adenocarcinoma,” “metastases to bone,” “metastases to liver” − 2 patiens each (0.1%), “breast cancer,” “gastric cancer,” “lymphoma,” “metastases to lung,” “metastases to lymph node,” “pancreatic cancer,” “kidney cancer,” “transitional cell carcinoma,” “metastases to peritoneal,” and “pancytopenia” − one patient each (0.1%). Among the outcomes of each event, six events recovered or improved, one event did not recover, one event was unknown, and six events resulted in death. Of the six deaths, five patients in the ND group included a “liver metastasis to liver” patient with a history of colon cancer and gastric cancer. “kidney cancer” and “metastases to lymph node” − one patient, “lymphoma” − one patient, “colon cancer,” “metastases to liver,” “metastases to peritoneal,” “metastases to lung” − one patient, “lung adenocarcinoma” and “metastases to bone” − one patient did not have a history or concurrent cancer. In the HD group, the single “metastases to bone” patient was associated with “colon cancer,” which was later resolved despite the occurrence of “metastases to bone.” This patient was also complicated by “lung adenocarcinoma” and “pulmonary malignant neoplasm.” The ADR incidence rate per 100 patient‐years was 0.4/100 patient‐years. Among 11 patients where ADRs related to cancers occurred, during the observation period (from the start of daprodustat treatment to 52 weeks or discontinuation), 7 patients (0.4%, *n* = 7/1634) were identified, while during the follow‐up period (a 52‐week follow‐up period after the observation period's conclusion), 5 patients (0.4%, *n* = 5/1406) were identified (one patient experienced ADRs related to cancers during both the observation and follow‐up periods).

The time to onset of each ADR of special interest is summarized in Table 4. In the safety population, the median (range) time to onset was 175 (7–283) days for thromboembolism (*n* = 7), 50 (8–207) days for hypertension (*n* = 9), 29 (21–37) days for CV events (*n* = 2), 261 (18–646) days for cancer (*n* = 11), 183 (105–261) days for retinal hemorrhage (*n* = 2), and 62 (62–62) days for GI erosion (*n* = 1).

Other ADRs of special interest included two patients (0.1%) of retinal hemorrhage, which was classified as serious but showed improvement, and one patient (0.1%) of gastrointestinal erosion—specifically serious diverticular perforation, which also improved. In patients with ADPKD (*n* = 58), no AE categorized as “progression of ADPKD” was reported during the observation period.

### 3.6 Other Contents Related to ADRs

In a multivariate analysis of baseline risk factors for ADRs, higher erythropoietin resistance index (ERI) during prior ESA treatment met the pre‐specified criteria in the categories “≥ 9.7–< 15.5 IU/week/kg/g/dL” and “≥ 15.5 IU/week/kg/g/dL” compared with the categories “without prior ESA treatment.” The adjusted odds ratios were 2.282 (95% CI, 1.043–4.991) for the “≥ 9.7–< 15.5 IU/week/kg/g/dL” group and 2.267 (95% CI, 1.181–4.351) for the “≥ 15.5 IU/week/kg/g/dL” group (Table 5). No consistent trend was observed between ADR occurrence and daprodustat dose across different ERIs (Table S2).

**TABLE 5:** Multivariate logistic regression analysis of risk factors for ADRs.

Item	Odds ratio	95% CI
Erythropoietin resistance index (ERI) of the previous treatment (ESA) [IU/week/kg/g/dL]		
< 5.7 vs. without prior ESA treatment	1.721	0.860–3.443
≥ 5.7–< 9.7 vs. without prior ESA treatment	1.240	0.520–2.956
≥ 9.7–< 15.5 vs. without prior ESA treatment	2.282	1.043–4.991
≥ 15.5 vs. without prior ESA treatment	2.267	1.181–4.351

*Note:* Multivariate logistic regression analysis: The number of patients for each candidate variable, the number of patients with ADRs, and the incidence proportion of ADRs are calculated. Based on the correlation results of each candidate variable, the proportion of missing values, and the distribution bias of patients among subgroups, variables to be used for multivariate analysis are determined, and multivariate analysis is conducted using a logistic regression model. Adjusted odds ratios and their 95% confidence intervals for each category are calculated. The adjusted odds ratio for ADR occurrence by patient background, identified using multivariate analysis, met the criterion of “asymptotic 95% confidence interval does not cross 1, and the point estimate exceeds 2 or is less than 0.5”.

The variables (at baseline) included gender, age, dialysis status, body mass index (BMI), comorbidity of thromboembolism, comorbidity of hypertension, comorbidity of cardiovascular events, comorbidity of cancer, comorbidity of retinal hemorrhage, comorbidity of hepatic dysfunction, comorbidity of heart failure, prior treatment of any iron, concomitant iron, Erythropoietin Resistance Index (ERI) of the previous treatment (ESA), hemoglobin.

Abbreviations: ADR, adverse drug reaction; ESA, erythropoiesis‐stimulating agent.

No consistent trend was observed between the rate of hemoglobin increase (g/dL per week) 4 weeks after daprodustat administration and the incidence of ADRs, as summarized in Table 6. The overall ADR rate varied across groups: 10.6% (7/66) in patients with a hemoglobin increase of ≥ 0.3–< 0.4 g/dL/week, 8.9% (5/56) in patients with a hemoglobin increase of ≥ 0.5–< 1 g/dL/week, and 8.5% (8/94) in patients with a hemoglobin increase of ≥ 0.2–< 0.3 g/dL/week. Notably, no ADRs were reported in patients with a hemoglobin increase of ≥ 1 g/dL/week (*n* = 5).

**TABLE 6:** Hemoglobin increase and ADRs.

	< 0.1 g/dL/week	≥ 0.1–< 0.2 g/dL/week	≥ 0.2–< 0.3 g/dL/week	≥ 0.3–< 0.4 g/dL/week	≥ 0.4–< 0.5 g/dL/week	≥ 0.5–< 1 g/dL/week	≥ 1 g/dL/week	Total
Safety population (*n*)	488	153	94	66	33	56	5	895
All ADRs, *n* (%)	27 (5.5)	8 (5.2)	8 (8.5)	7 (10.6)	2 (6.1)	5 (8.9)	0 (0.0)	57 (6.4)
Serious ADRs, *n* (%)	12 (2.5)	3 (2.0)	3 (3.2)	3 (4.5)	1 (3.0)	1 (1.8)	0 (0.0)	23 (2.6)
ND group (*n*)	219	92	65	45	23	28	3	475
All ADRs, *n* (%)	10 (4.6)	6 (6.5)	6 (9.2)	4 (8.9)	0 (0.0)	2 (7.1)	0 (0.0)	28 (5.9)
Serious ADRs, *n* (%)	5 (2.3)	2 (2.2)	2 (3.1)	3 (6.7)	0 (0.0)	1 (3.6)	0 (0.0)	13 (2.7)
PD group (*n*)	42	6	6	4	4	5	2	69
All ADRs, *n* (%)	2 (4.8)	0 (0.0)	0 (0.0)	0 (0.0)	2 (50.0)	0 (0.0)	0 (0.0)	4 (5.8)
Serious ADRs, *n* (%)	0 (0.0)	0 (0.0)	0 (0.0)	0 (0.0)	1 (25.0)	0 (0.0)	0 (0.0)	1 (1.4)
HD group (*n*)	226	55	23	16	6	23	0	349
All ADRs, *n* (%)	14 (6.2)	2 (3.6)	2 (8.7)	2 (12.5)	0 (0.0)	3 (13.0)	0 (0.0)	23 (6.6)
Serious ADRs, *n* (%)	6 (2.7)	1 (1.8)	1 (4.3)	0 (0.0)	0 (0.0)	0 (0.0)	0 (0.0)	8 (2.3)

Abbreviations: ADR, adverse drug reaction; HD, hemodialysis; ND, no dialysis; PD, peritoneal dialysis.

There were no clear trends observed between daprodustat dose and the incidence of ADRs (Table S3).

Figure 2 illustrates the changes in hemoglobin levels over time during the observation period, while subgroup analyses stratified by prior ESA treatment are presented in Figure S2. The trend of the average hemoglobin values showed no signs of overshooting.

**FIGURE 2:**
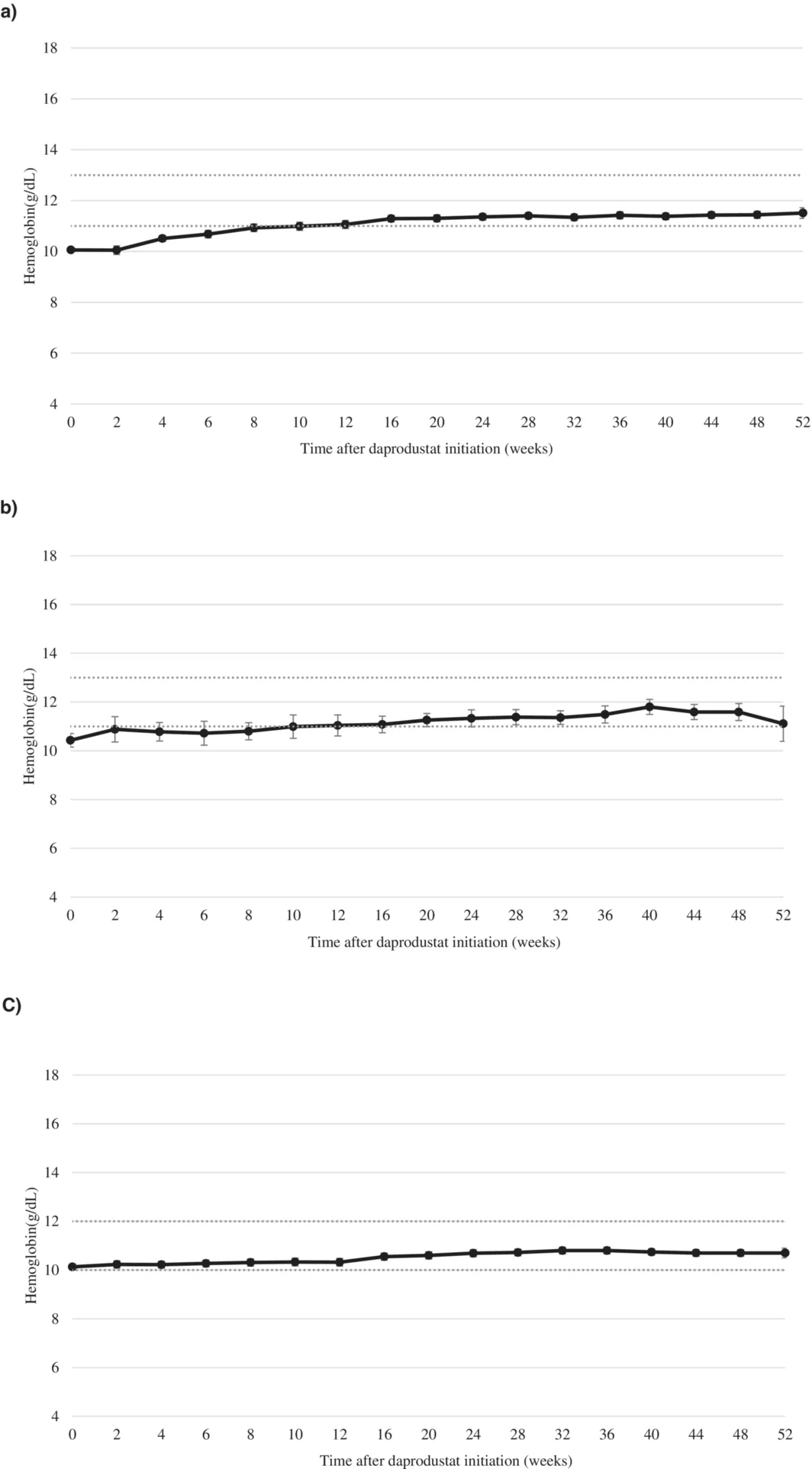
Hemoglobin over time. (A) All ND patients (*n* = 1048). (B) All PD patients (*n* = 116). (C) All HD patients (*n* = 470).

## 4 Discussion

This final analysis of a postmarketing surveillance study conducted in Japan aimed to evaluate the safety of daprodustat in routine clinical practice. The overall incidence proportion of ADRs was reported at 5.6%. Patients undergoing dialysis had a higher incidence of ADRs (PD: 6.9%; HD: 6.6%) compared to those in the ND group (4.8%). ADRs of special interest—such as thromboembolism, hypertension, CV events, cancer, and retinal hemorrhage—were observed in less than 1% of patients overall and across dialysis status subgroups. No ADRs associated with ADPKD progression in patients with ADPKD were observed. No consistent trend was identified between the rate of hemoglobin rise (g/dL per week) and ADRs. No new safety concerns emerged during the analysis.

The overall incidence proportion of ADRs was 5.6%, which did not exceed the results of three Japan Phase III studies targeting patients receiving ND, PD, and HD, where the incidence proportion was 7% [19, 20, 21, 23]. This may be attributed to differences in the use of daprodustat in real‐world settings compared to clinical trial settings, such as differences in patient selection or more conservative dosing (the median daily dose of daprodustat: 4.0 mg/day in this study, 4.6 mg/day in three Japan Phase III studies). Despite these factors, no new safety concerns were observed in this study. Additionally, dialysis patients (PD 6.9%, HD 6.6%) showed a higher incidence proportion of ADRs reports compared to the nondialysis group (ND 4.8%). However, this trend was similar to the results of the three Japan Phase III studies targeting patients receiving ND, PD, and HD (PD 14%, HD 6%–7%, ND 6%).

Regarding cancers, in three Japanese Phase III trials targeting patients undergoing ND, PD, and HD, the incidence rate per patient year was 1.53 for patients treated with ESA and 1.28 for those treated with daprodustat. Additionally, in the global ASCEND program, an increase in cancers was observed in the daprodustat group among patients undergoing ND [25, 26]. However, post hoc analysis indicated that daprodustat was not associated with an increased risk of cancer compared to ESA among patients undergoing ND or HD [27]. The incidence rate of ADRs related to cancers observed in the interim analysis of the present study (0.4 per patient year) did not exceed the results of the three Japanese Phase III trials. However, the results of the final analysis were anticipated, as the number of patients followed up for up to 2 years was 159 in the interim analysis [24]. In the final analysis, with 1406 patients observed over a maximum follow‐up period of 2 years, the incidence rate remained at 0.4 per patient year, showing no increase from the interim analysis rate (0.4). Furthermore, the incidence proportions during the observation and follow‐up periods were both 0.4%. This may be due to differences in the actual use of daprodustat compared to clinical trial settings, such as differences in patient selection or more conservative dosing. Additionally, in real‐world usage situations, the frequency of screenings may be lower than in prospective interventional studies, potentially increasing the likelihood of undetected cases of cancers. Notably, this study did not identify any specific trends regarding the types or locations of cancers. Despite these factors, no new safety concerns for cancers were observed in this study.

In this study, the incidence of thromboembolism as an ADR was less than 1% overall (0.4% overall; ND: 0.4%, PD: 0.0%, HD: 0.6%), with AEs reported at 1.5%. In Japanese Phase III trials, the incidence of thromboembolism‐related AEs was 5% (17/369 patients) in the daprodustat group and 6% (16/285 patients) in the control group (ESA group). The incidence of thromboembolism observed in this study did not exceed the rates reported in the Japanese Phase III trials. In this study, as ADRs related to CV events (except thromboembolism), only two ADRs related to heart failure or worsening heart failure were reported: one patient of worsening heart failure in the ND group and one initial heart failure event in the HD group. No other CV events (except thromboembolism) were reported as ADRs. Moreover, the incidence proportion of AEs related to heart failure was 1.7% (28/1634 patients) in this study. Regarding heart failure, the incidence frequency of AEs related to heart failure in the three Phase III trials conducted in Japan was 1.6% (6/369 patients) in the study daprodustat and 3.5% (10/285 patients) in the control group (ESA group). The incidence proportion of AEs related to heart failure observed in the final analysis of this study was comparable to the results of the three Phase III trials conducted in Japan.

Regarding other ADRs of special interest, the incidence proportion of ADRs for hypertension in this study was less than 1% (overall: 0.6%, ND: 0.6%, PD: 0.0%, HD: 0.4%), and AEs were observed at 1.9%. In three Japanese Phase III trials, the incidence proportions of AEs for hypertension and increased blood pressure were 4% and 2%, respectively, in the daprodustat group, and 6% and 2%, respectively, in the control group (ESA group). The incidence proportion of hypertension observed in this study did not exceed the incidence proportions observed in the three Japanese phase III trials. Moreover, no ADRs associated with ADPKD progression in patients with ADPKD were observed, with ADRs for retinal hemorrhage reported at 0.1% (0.2% as AEs); and no new retinal hemorrhage ADRs were observed. These rates did not exceed the incidence proportions reported in the Japanese Phase III trials. The incidence proportion of ADRs related to gastrointestinal erosion was also low (0.1%).

Regarding ADRs, in a multivariate analysis of baseline risk factors for ADRs, patients with higher ERI during prior ESA treatment—specifically in the pre‐specified ranges ≥ 9.7–< 15.5 and ≥ 15.5 IU/week/kg/g/dL—met the study's pre‐specified criteria compared with patients without prior ESA treatment. However, the types of ADRs showed a similar trend across each category, and all observed ADRs fell within the safety profile of daprodustat. It has been noted that a high ERI may influence prognosis [28], making it challenging to evaluate unequivocally, but it was considered that the patient's condition could potentially contribute to the increased incidence of ADRs. Additionally, no consistent trend was observed between the incidence proportion of ADRs and the dosing of daprodustat by ERI, suggesting that the likelihood of additional risks arising from the pharmacological effects of daprodustat is low. Regarding ADRs, the correlations with the rate of hemoglobin increase (g/dL per week) 4 weeks after daprodustat administration or the daprodustat dose were also examined. Although a significant conclusion could not be drawn due to inconsistencies in patient characteristics between groups and an insufficient number of patients in groups with particularly high hemoglobin increase rates (g/dL/week) or high doses of daprodustat, no consistent trend was observed with the incidence of ADRs in either correlation.

This study has several inherent limitations due to its observational design. Notably, the lack of a control group precluded comparisons of daprodustat's safety relative to other treatments, and safety outcomes for patients who discontinued treatment during the 52‐week follow‐up period were only collected while they were still undergoing treatment. Although information on pre‐ and concomitant medications was reported, data on patient adherence to prescribed doses were unavailable. Additional limitations include restrictions on data collection via EDC, as well as the potential for nonresponses or limited/inaccurate responses from patients. The logistic regression model used in the analysis was based on the assumption that the collected data were accurate and representative; however, potential bias in case collection may have influenced the results. Selection bias cannot be excluded because treatment choice was made in routine clinical practice. Patients at higher baseline risk for thrombosis, malignancy, or other clinically important outcomes may have been less likely to receive daprodustat, which may have affected the observed incidence of AEs. Furthermore, some patients received daprodustat at doses lower than the recommended starting dose, which could have affected the incidence proportion of ADRs.

In conclusion, in this final analysis of an observational study on daprodustat safety conducted in Japan, no new safety concerns were observed in real‐world clinical practice, including no observed increase in cancer‐related events during the follow‐up period.

## Funding

Funding for this study (GSK Study 214119) was provided by GlaxoSmithKline (GSK).

## Ethics Statement

This study was approved by the Ethics Review Board of Kitamachi Clinic, which acted as the central ethics committee. The study was carried out in accordance with the Ministry of Health, Labour and Welfare (MHLW) Ordinance No. 171, issued on December 20, 2004, which establishes Good Postmarketing Surveillance Practice (GPSP) guidelines for conducting postmarketing surveillance and studies on pharmaceuticals.

## Consent

Prior to participation, all patients provided written informed consent.

## Conflicts of Interest

Tadao Akizawa reports receiving consulting and lecture fees from Kyowa Kirin, Kyomi Kanaya, Kiyomi Aizawa, Masanori Onoki, Hirofumi Ozeki, Kazuko Suzuki, Ai Hayashi, and Tomohiko Asamura are employees of GSK. Kazuko Suzuki holds stock in GSK.

## Supporting information


**Table S1:** Heart failure‐related ADRs.
**Table S2:** ADRs based on daprodustat dose across different ERIs.
**Table S3:** ADRs based on daprodustat dose.

**Figure S1:** Daprodustat dosing.

**Figure S2:** Hemoglobin over time: subgroup analyses stratified by prior ESA treatment.

## Data Availability

The anonymized individual patient data from this study are not available to the public, as the contract with the study site does not permit data sharing with third parties, excluding health authorities. The related documents for this study can be found at www.gsk‐studyregister.com/en.
