# Supplementary material for: Safety of Daprodustat for the Treatment of Chronic Kidney Disease Anemia: Final Analysis of a Multicenter Postmarketing Surveillance Study in Japan
Source: Ther Apher Dial. 2026 Jul 6;30(6):908–24. doi: 10.1002/1744-9987.70182 (PMC13738054; doi:10.1002/1744-9987.70182)
Supplement: Supplementary file 1 [file TAP-30-908-s001.pdf]

Supplemental Figure 2. Hemoglobin over time: subgroup analyses stratified by prior ESA treatment

A) ND patients with prior injectable ESA treatment (n=354)

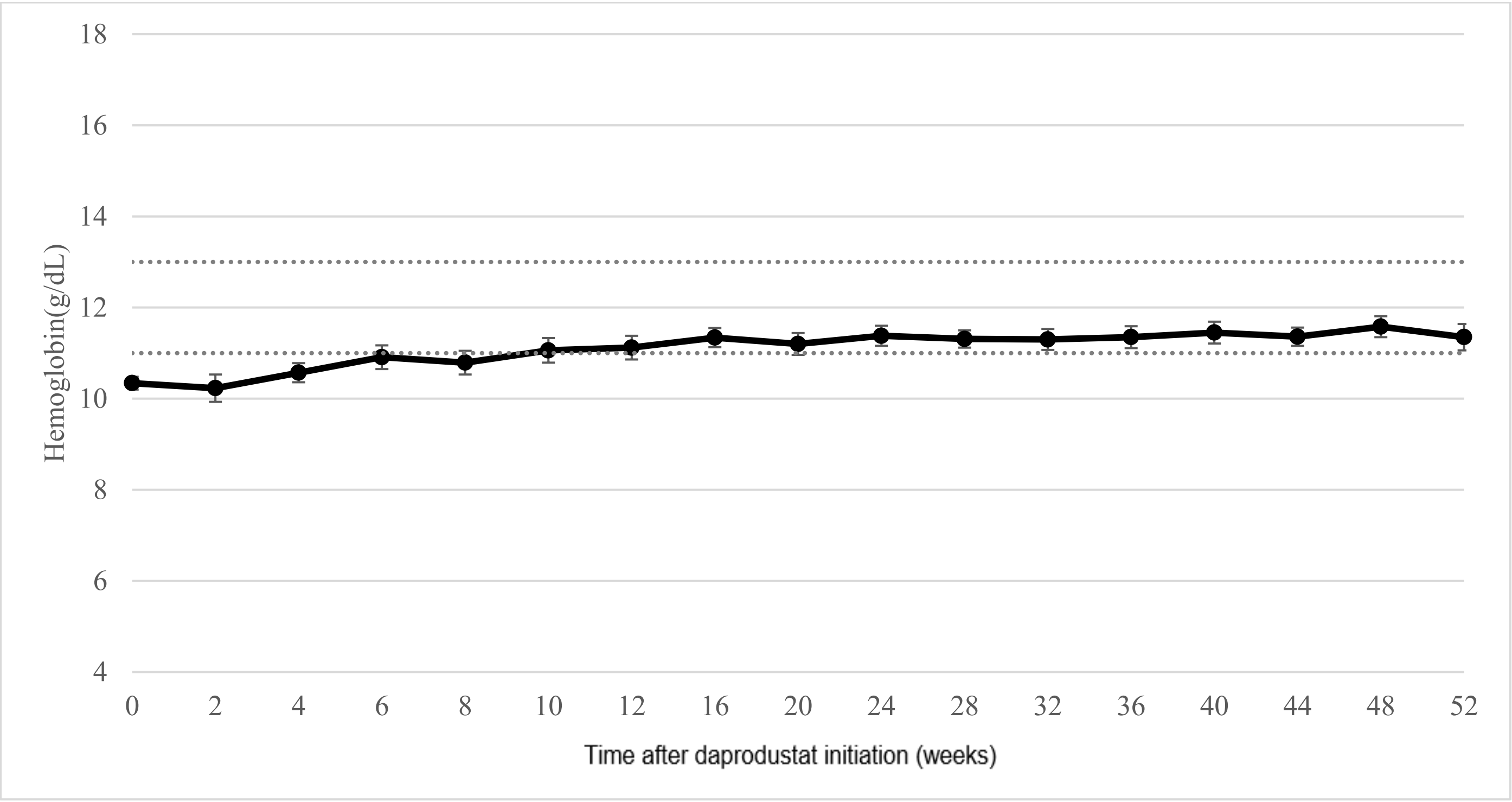

Critical & Sensitive Information (CSI)

B) ND patients without prior injectable ESA or HIF-PHI treatment (n=670)

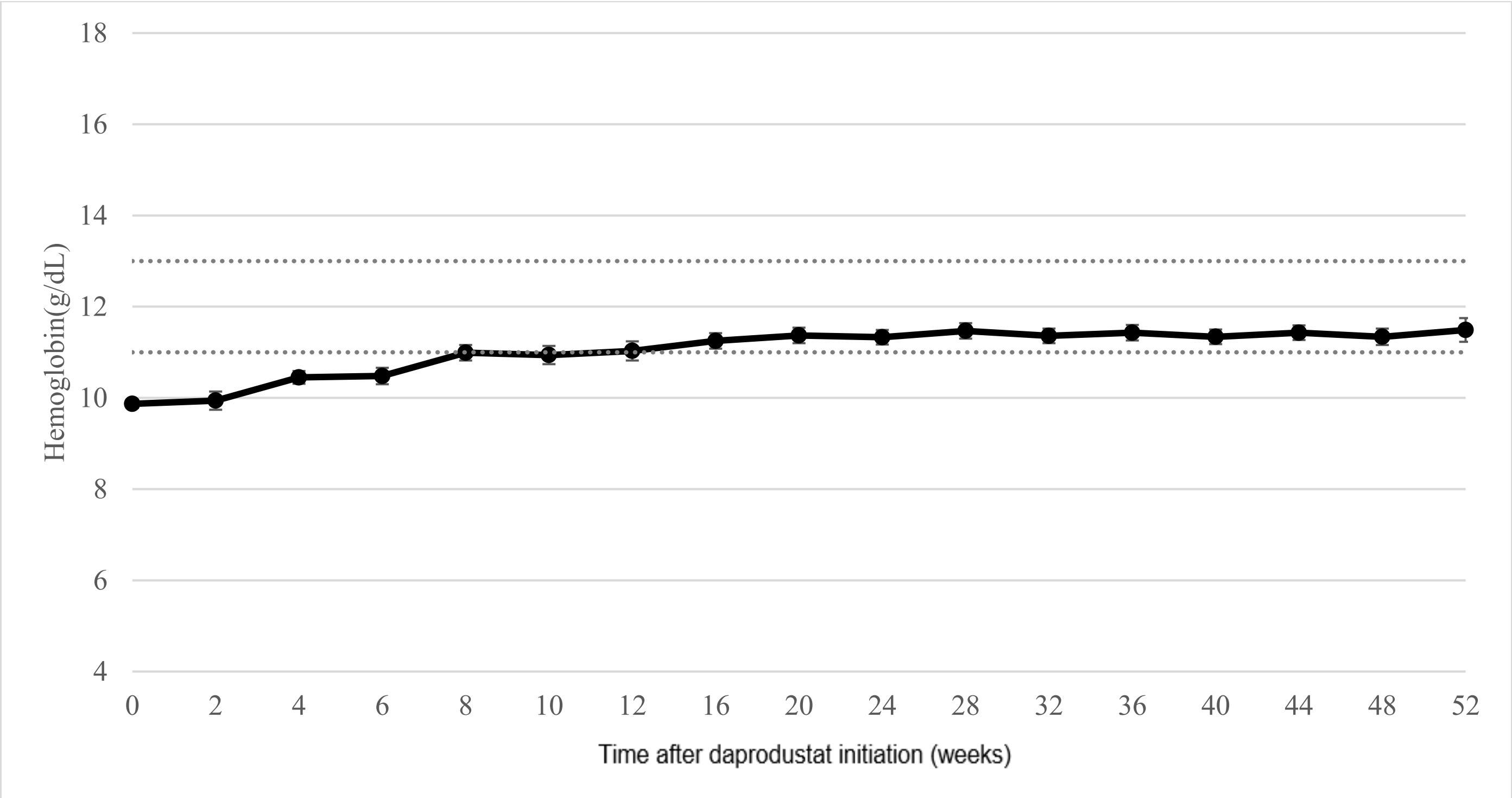

Critical & Sensitive Information (CSI)

C) PD patients with prior injectable ESA treatment (n=84)

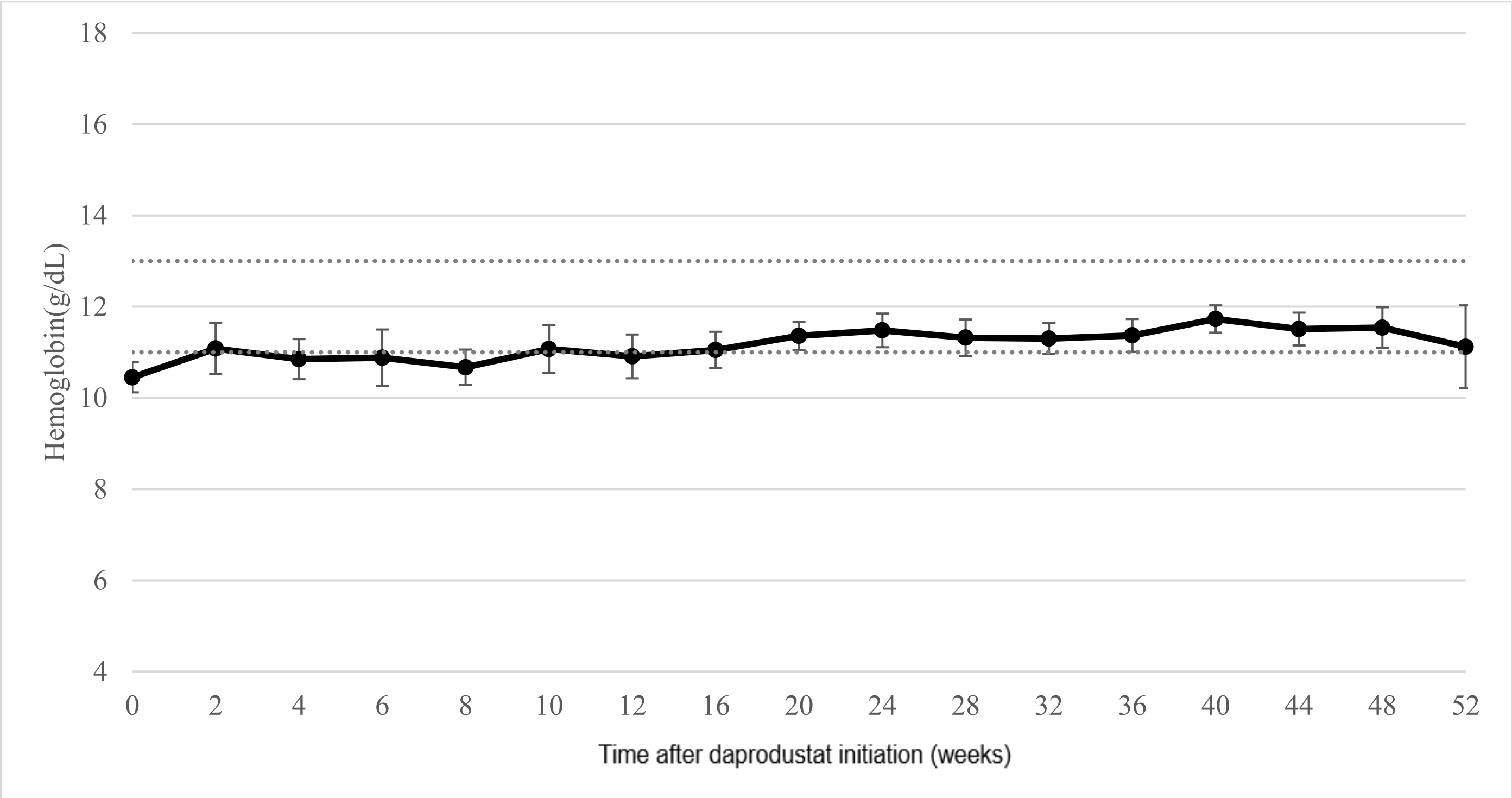

Critical & Sensitive Information (CSI)

D) PD patients without prior injectable ESA or HIF-PHI treatment (n=25)

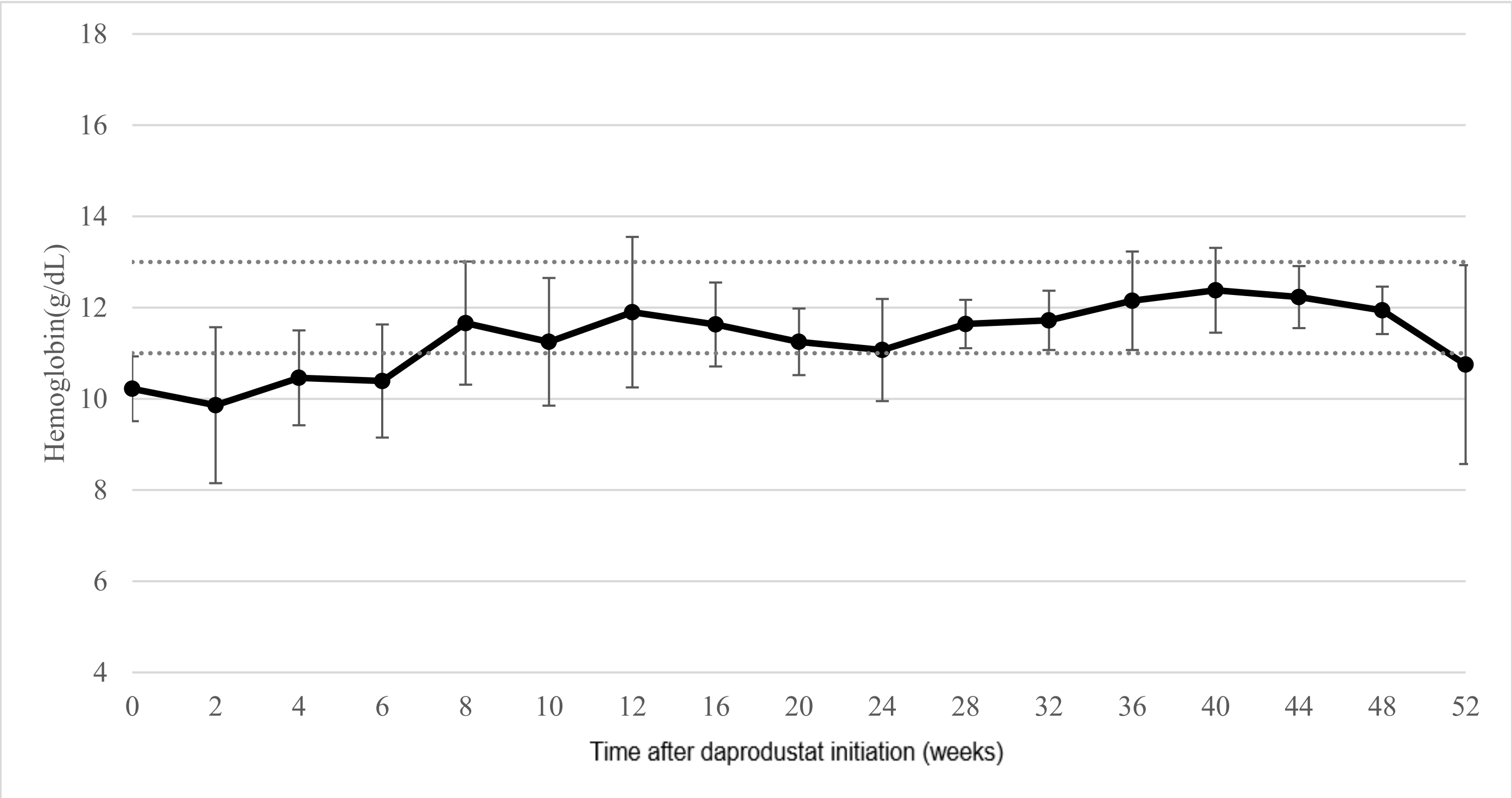

Critical & Sensitive Information (CSI)

E) HD patients with prior injectable ESA treatment (n=291)

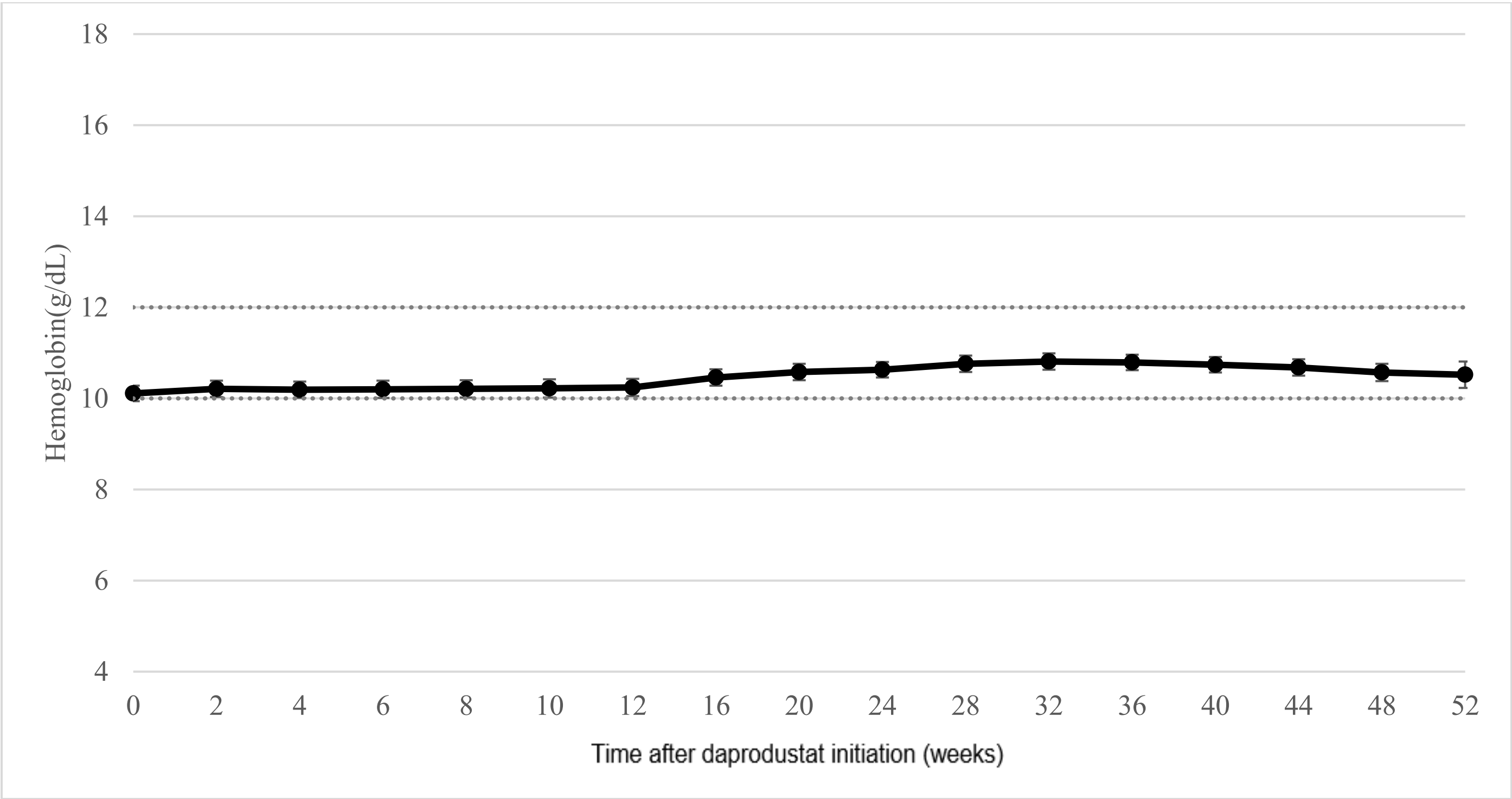

Critical & Sensitive Information (CSI)

F) HD patients without prior injectable ESA or HIF-PHI treatment (n=134)

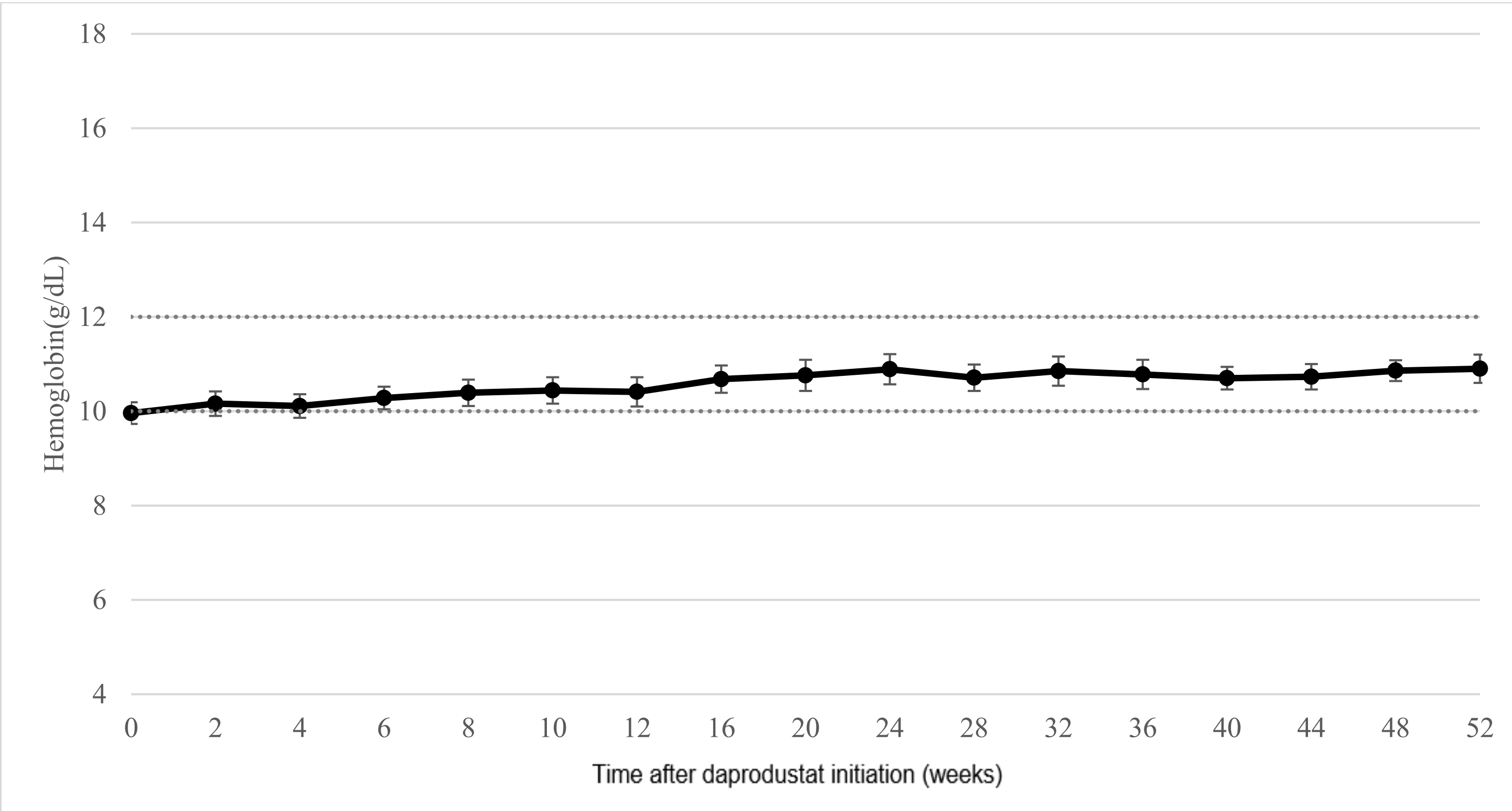

Critical & Sensitive Information (CSI)
