# Supplementary material for: Safety of Daprodustat for the Treatment of Chronic Kidney Disease Anemia: Final Analysis of a Multicenter Postmarketing Surveillance Study in Japan
Source: Ther Apher Dial. 2026 Jul 6;30(6):908–24. doi: 10.1002/1744-9987.70182 (PMC13738054; doi:10.1002/1744-9987.70182)
Supplement: Supplementary file 3 [file TAP-30-908-s003.docx]

# Supplementary Material

# Supplemental Tables

**Supplemental Table 1. Heart Failure–related ADRs**

| **Dialysis status at baseline** | **Preexisting HF** | **No prior HF** |
| --- | --- | --- |
| **ND** | **N=178** | **N=870** |
| ADR of HF or exacerbation of HF, n (%) | 1 (0.6) | 0 (0.0) |
| **PD** | **N=24** | **N=92** |
| ADR of HF or exacerbation of HF, n (%) | 0 (0.0) | 0 (0.0) |
| **HD** | **N=47** | **N=423** |
| ADR of HF or exacerbation of HF, n (%) | 0 (0.0) | 1 (0.2) |

ADR, adverse drug reaction; HD, hemodialysis; HF, heart failure; ND, no dialysis; PD, peritoneal dialysis.

**Supplemental Table 2. ADRs based on daprodustat dose across different ERIs**

|  | **Safety population**  **(N=1634)** | | **Without prior ESA treatment**  **(n=905)** | | **ERI**  **˂5.7**  **(n=185)** | | **ERI**  **≥5.7 to ˂9.7**  **(n=140)** | | **ERI**  **≥9.7 to ˂15.5**  **(n=120)** | | **ERI**  **≥15.5**  **(n=234)** | |
| --- | --- | --- | --- | --- | --- | --- | --- | --- | --- | --- | --- | --- |
|  | **Pts,**  **N** | **ADR,**  **n (%)** | **Pts,**  **n** | **ADR,  n (%)** | **Pts,**  **N** | **ADR,  n (%)** | **Pts,**  **N** | **ADR,  n (%)** | **Pts,**  **n** | **ADR,  n (%)** | **Pts,**  **n** | **ADR,  n (%)** |
| Total initial daily dose, mg |  |  |  |  |  |  |  |  |  |  |  |  |
| ˂2 | 36 | 1  (2.8) | 26 | 1  (3.8) | 10 | 0  (0.0) | 0 | 0  (-) | 0 | 0  (-) | 0 | 0  (-) |
| 2 | 584 | 25 (4.3) | 475 | 18  (3.8) | 46 | 4  (8.7) | 22 | 0  (0.0) | 12 | 0  (0.0) | 16 | 3  (18.8) |
| >2 to ˂4 | 2 | 0  (0.0) | 1 | 0  (0.0) | 0 | 0  (-) | 1 | 0  (0.0) | 0 | 0  (-) | 0 | 0  (-) |
| 4 | 988 | 64 (6.5) | 388 | 16  (4.1) | 129 | 10  (7.8) | 116 | 8  (6.9) | 107 | 10  (9.3) | 216 | 18  (8.3) |
| >4 | 24 | 1  (4.2) | 15 | 1  (6.7) | 0 | 0  (-) | 1 | 0  (0.0) | 1 | 0  (0.0) | 3 | 0  (0.0) |
| Average daily dose, mg/day |  |  |  |  |  |  |  |  |  |  |  |  |
| ˂2 | 78 | 4  (5.1) | 53 | 3  (5.7) | 17 | 1  (5.9) | 2 | 0  (0.0) | 2 | 0  (0.0) | 4 | 0  (0.0) |
| ≥2 to ˂4 | 694 | 31 (4.5) | 505 | 21  (4.2) | 76 | 5  (6.6) | 40 | 1  (2.5) | 20 | 1  (5.0) | 36 | 3  (8.3) |
| ≥4 to ˂6 | 644 | 40 (6.2) | 276 | 9  (3.3) | 77 | 8  (10.4) | 80 | 4  (5.0) | 72 | 4  (5.6) | 116 | 13  (11.2) |
| ≥6 to ˂8 | 118 | 12 (10.2) | 33 | 2  (6.1) | 14 | 0  (0.0) | 9 | 2  (22.2) | 16 | 4  (25.0) | 42 | 4  (9.5) |
| ≥8 to ˂12 | 74 | 1  (1.4) | 30 | 1  (3.3) | 0 | 0  (-) | 8 | 0  (0.0) | 7 | 0  (0.0) | 27 | 0  (0.0) |
| ≥12 to ˂16 | 23 | 3 (13.0) | 7 | 0  (0.0) | 1 | 0  (0.0) | 1 | 1  (100.0) | 3 | 1  (33.3) | 9 | 1  (11.1) |
| ≥16 to ≤24 | 3 | 0  (0.0) | 1 | 0  (0.0) | 0 | 0  (-) | 0 | 0  (-) | 0 | 0  (-) | 1 | 0  (0.0) |
| >24 | 0 | 0  (-) | 0 | 0  (-) | 0 | 0  (-) | 0 | 0  (-) | 0 | 0  (-) | 0 | 0  (-) |
| Maximum daily dose, mg |  |  |  |  |  |  |  |  |  |  |  |  |
| ˂2 | 20 | 0  (0.0) | 17 | 0  (0.0) | 3 | 0  (0.0) | 0 | 0  (-) | 0 | 0  (-) | 0 | 0  (-) |
| ≥2 to ˂4 | 438 | 17  (3.9) | 375 | 15  (4.0) | 32 | 2  (6.3) | 10 | 0  (0.0) | 8 | 0  (0.0) | 5 | 0  (0.0) |
| ≥4 to ˂6 | 661 | 41 (6.2) | 316 | 8  (2.5) | 105 | 8  (7.6) | 72 | 5  (6.9) | 50 | 4  (8.0) | 95 | 14  (14.7) |
| ≥6 to ˂8 | 227 | 13 (5.7) | 97 | 10  (10.3) | 25 | 2  (8.0) | 29 | 0  (0.0) | 29 | 0  (0.0) | 40 | 1  (2.5) |
| ≥8 to ˂12 | 167 | 12 (7.2) | 62 | 3  (4.8) | 17 | 2  (11.8) | 17 | 1  (5.9) | 16 | 4  (25.0) | 50 | 2  (4.0) |
| ≥12 to ˂16 | 88 | 4  (4.5) | 30 | 0  (0.0) | 3 | 0  (0.0) | 9 | 1  (11.1) | 11 | 0  (0.0) | 30 | 3  (10.0) |
| ≥16 to ≤24 | 33 | 4 (12.1) | 8 | 0  (0.0) | 0 | 0  (-) | 3 | 1  (33.3) | 6 | 2  (33.3) | 15 | 1  (6.7) |
| >24 | 0 | 0  (-) | 0 | 0  (-) | 0 | 0  (-) | 0 | 0  (-) | 0 | 0  (-) | 0 | 0  (-) |

ADR, adverse drug reaction; ERI, Erythropoietin Resistance Index; Pts, Patients.

**Supplemental Table 3. ADRs based on daprodustat dose**

|  | **Safety population**  **(N=1634)** | | **ND**  **(n=1048)** | | **PD**  **(n=116)** | | **HD**  **(n=470)** | |
| --- | --- | --- | --- | --- | --- | --- | --- | --- |
|  | **Patients,**  **n** | **ADR,**  **n (%)** | **Patients,**  **n** | **ADR,  n (%)** | **Patients,**  **n** | **ADR,  n (%)** | **Patients,**  **n** | **ADR,  n (%)** |
| Total initial daily dose, mg |  |  |  |  |  |  |  |  |
| ˂2 | 36 | 1 (2.8) | 26 | 0 (0.0) | 4 | 0 (0.0) | 6 | 0 (0.0) |
| 2 | 584 | 25 (4.3) | 540 | 22 (4.1) | 17 | 0 (0.0) | 27 | 3 (11.1) |
| ˃2 to ˂4 | 2 | 0 (0.0) | 0 | 0 (-) | 0 | 0 (-) | 2 | 0 (0.0) |
| 4 | 988 | 64 (6.5) | 467 | 27 (5.8) | 94 | 8 (8.5) | 427 | 28 (6.6) |
| ˃4 | 24 | 1 (4.2) | 15 | 1 (6.7) | 1 | 0 (0.0) | 8 | 0 (0.0) |
| Average daily dose, mg/day |  |  |  |  |  |  |  |  |
| ˂2 | 78 | 4 (5.1) | 55 | 2 (3.6) | 5 | 0 (0.0) | 18 | 1 (5.6) |
| ≥2 to ˂4 | 694 | 31 (4.5) | 578 | 23 (4.0) | 30 | 1 (3.3) | 86 | 6 (7.0) |
| ≥4 to ˂6 | 644 | 40 (6.2) | 358 | 19 (5.3) | 51 | 5 (9.8) | 239 | 16 (6.7) |
| ≥6 to ˂8 | 118 | 12 (10.2) | 32 | 6 (18.8) | 22 | 2 (9.1) | 62 | 4 (6.5) |
| ≥8 to ˂12 | 74 | 1 (1.4) | 19 | 0 (0.0) | 6 | 0 (0.0) | 47 | 1 (2.1) |
| ≥12 to ˂18 | 23 | 3 (13.0) | 5 | 0 (0.0) | 2 | 0 (0.0) | 16 | 3 (18.8) |
| ≥18 to ≤24 | 3 | 0 (0.0) | 1 | 0 (0.0) | 0 | 0 (-) | 2 | 0 (0.0) |
| ˃24 | 0 | 0 (-) | 0 | 0 (-) | 0 | 0 (-) | 0 | 0 (-) |
| Maximum daily dose, mg |  |  |  |  |  |  |  |  |
| ˂2 | 20 | 0 (0.0) | 15 | 0 (0.0) | 3 | 0 (0.0) | 3 | 0 (0.0) |
| ≥2 to ˂4 | 438 | 17 (3.9) | 417 | 15 (3.6) | 10 | 0 (0.0) | 12 | 1 (8.3) |
| ≥4 to ˂6 | 661 | 41 (6.2) | 433 | 24 (5.5) | 43 | 5 (11.6) | 193 | 12 (6.2) |
| ≥6 to ˂8 | 227 | 13 (5.7) | 104 | 5 (4.8) | 24 | 1 (4.2) | 97 | 6 (6.2) |
| ≥8 to ˂12 | 167 | 12 (7.2) | 49 | 3 (6.1) | 26 | 2 (7.7) | 87 | 7 (8.0) |
| ≥12 to ˂18 | 88 | 4 (4.5) | 26 | 3 (11.5) | 8 | 0 (0.0) | 52 | 1 (1.9) |
| ≥18 to ≤24 | 33 | 4 (12.1) | 4 | 0 (0.0) | 2 | 0 (0.0) | 26 | 4 (15.4) |
| ˃24 | 0 | 0 (-) | 0 | 0 (-) | 0 | 0 (-) | 0 | 0 (-) |

ADR, adverse drug reaction; HD, hemodialysis; ND, no dialysis; PD, peritoneal dialysis.
